# Mental Health and Mental Health Care in Iran: Addressing Social Inequalities

**DOI:** 10.3390/healthcare13233131

**Published:** 2025-12-01

**Authors:** Saeid Zandi, Farnoosh Oghani-Esfahani, Fereshteh Ahmadi, Roqayyeh Sabbaghi-Dehkalani, Sharareh Akhavan

**Affiliations:** 1 Department of Social Work and Criminology, Faculty of Health and Occupational Studies, University of Gävle, 801 76 Gävle, Sweden; 2 Department of Counseling, Faculty of Psychology and Education, Allameh Tabataba’i University, Tehran 1489684511, Iran; oghanifarnoosh2@gmail.com; 3 Department of Social Work, Criminology, and Public Health Sciences, Faculty of Health and Occupational Studies, University of Gävle, 801 76 Gävle, Sweden; fereshteh.ahmadi@hig.se; 4 Department of Clinical Psychology, Faculty of Medicine, Zanjan University of Medical Sciences, Zanjan 4513956184, Iran; r.sabbaghi.deh@gmail.com; 5 Department of Social Work and Psychology, Faculty of Health and Occupational Studies, University of Gävle, 801 76 Gävle, Sweden; sharareh.akhavan@hig.se

**Keywords:** equity in health, health disparities, healthcare access, healthcare system, mental disorders, mental health services, primary health care, public health, socioeconomic factors, systematic review

## Abstract

**Background/Objectives**: Iran carries a significant burden of mental health disorders. This study aimed to describe the status of mental health and mental health care in Iran between 2012 and 2023, addressing inequalities and mapping existing challenges in the mental health care system. **Methods**: A systematic literature review was conducted. Databases including Medline, CINAHL, APA PsycINFO, Scopus, Web of Science, and the Cochrane Library, as well as local databases such as SID, Magiran, and Noormags, were searched to identify studies related to mental health care in Iran. A total of 59 studies met the inclusion criteria. An inductive approach and thematic analysis were used to synthesize themes from the data. **Results**: Lower socioeconomic status (SES) was associated with higher rates of mental disorders due to poverty-related stressors and limited access to quality care. Gender disparities revealed that women are more vulnerable to mental health problems, exacerbated by perceived gender inequality. Ethnic minorities and undocumented migrant populations faced inadequate healthcare services, resulting in poorer mental health outcomes. Children and older adults also experienced mental health challenges influenced by sociodemographic factors. The main challenge for mental health care is establishing mechanisms to ensure more equitable access for all citizens. Additional challenges include limited awareness among policymakers, insufficient budget allocation, weak prevention programs, and poor intra- and inter-sectoral coordination and collaboration. A shortage of mental health care providers, as well as deficiencies in structure, system processes, and resources, further hinder progress. **Conclusions**: Socioeconomic factors exacerbate the challenges of Iran’s under-resourced mental health system. To address these issues, equity considerations must be integrated into mental health policies. Key interventions include the routine monitoring of mental health indicators, expanding insurance coverage for mental health services, and establishing dedicated services for children.

## 1. Introduction

Mental health is a crucial aspect of human health and well-being, and its importance has been increasingly acknowledged, especially in the context of achieving global development goals (Goal 3 in particular, which includes health and well-being for all ages). Mental health is a fundamental human right, enabling individuals to connect, function, cope, and thrive [1]. People who are more exposed to unfavorable circumstances, such as conflict, disease outbreaks, social injustice, gender and ethnic inequalities, and socio-economic disadvantages, as well as poor access to health care services, are at higher risk of experiencing mental health disorders (MHDs). As previous research shows, health is shaped by access to power and resources based on sex, gender identity, and sexual orientation, as well as other intersecting social categories [2]. Risks to mental health manifest themselves at all stages in life. Mental health care services must use a life-course approach [3]. In this study, we include the social, economic, and environmental factors that influence an individual’s mental health status, such as poverty, education, housing, employment, social support networks, community safety, and exposure to violence or discrimination as social determinants of mental health. The emphasis is on how these factors impact the mental health of individuals and/or populations.

The social, economic, and systemic factors that affect access to, quality of, and outcomes from mental health care services are social determinants of mental health care. These include the availability of mental health services, healthcare policies, insurance coverage, transportation to healthcare facilities, cultural competence of healthcare providers, and stigma associated with seeking mental health care. Here, focus is on how these factors influence the ability of individuals to receive effective mental health treatment and support. Iran is one of the countries in the world where the percentage of MHDs is almost double the world average [4,5,6]. As the second-largest Middle Eastern country with a population of approximately 89 million, there appears to be a steep increase in the prevalence of mental disorders in Iran [4]. The health disease burden data based on the Disability Adjusted Life Years (DALYs) index shows that in Iran, since 2014, mental patients have been ranked second among 22 disease groups, and the DALYs data from 2018 revealed that mental diseases accounted for 10.31% of the total health disease burden. In Iran, four national surveys were conducted in the field of measuring mental disorders and problems, and the latest study showed that 23.4% of Iranian people suffer from psychological problems [7].

In the broader Middle Eastern context, studies show that fragmented healthcare systems, workforce shortages, and uneven resource allocation, particularly between urban and rural areas, limit access to mental health services [8,9]. Social and cultural factors such as stigma, mental health illiteracy, and the reliance on family or traditional healers emerge as barriers to professional help-seeking [10,11]. Studies from Türkiye provide detailed evidence of socioeconomic and demographic inequalities in mental health. Lower income and educational attainment are consistently linked to poorer mental health outcomes and lower service utilization [12,13]. Broader national analyses confirm that cost-related unmet needs disproportionately affect women, low-income individuals, and those with language barriers [14]. These findings align with broader Middle Eastern trends, emphasizing the role of social and economic disadvantage in shaping social inequalities in mental health outcomes and access to mental health care.

In this study, we use the theory of social determinants of health [15] and a model about the social basis of disparities in health [16] to describe the status of mental health and mental health care in Iran (2012–2023) by addressing the social inequalities. The theory of social determinants of health explains why social and economic conditions increase the mental health burden. The social basis of disparities in health model explains how structural inequalities create gaps in care and outcomes. Together, they provide a theoretical basis for understanding how structural and social inequalities drive elevated rates of mental health disorders and inequitable access to care.

Moreover, we will address the existing challenges of mental health care and evaluate suggested policy points for the improvement of mental health care in this country. One of the key research gaps in Iran’s mental health care system lies in understanding how social inequalities and systemic barriers restrict access to services and what interventions can effectively overcome these obstacles. Addressing these gaps could significantly improve early detection, treatment continuity, and equity in mental health service delivery across the country.

We sought to answer the following research questions: (1) What is the state of mental health and mental health care policy and resource allocation in Iran? (2) What disparities exist in the distribution of mental health facilities and human resources? (3) What disparities exist in accessing mental health services for vulnerable and underserved populations? (4) How can the health care system in Iran bridge the mental healthcare gap?

This review contributes to the existing literature on Iranian mental health policy and service accessibility by highlighting how social inequalities shape access to care and by exploring approaches to improve service equity and effectiveness.

Based on the applied model, inequitable and suboptimal health outcomes arise through several interconnected pathways, including processes of social stratification, unequal patterns of exposure to risks, variations in susceptibility, and the broader social impacts that follow ill health. These mechanisms interact over time, and the accumulation of socially structured disadvantages plays a pivotal role in shaping health outcomes and ultimately generating health inequities [16].

Clarifying what is meant by health and health inequalities is essential for establishing a common language among researchers, policymakers, and practitioners. In this article, we use the term health inequalities to refer to patterned differences in health outcomes that are systematic, preventable, and unjust—whether these differences occur between population groups, within subgroups of the same population, or along a gradient defined by social position [17].

## 2. Methods

We conducted a systematic review according to PRISMA guidelines [18] to map the status of mental health and mental health care in Iran, confronting social inequality, and also to address the existing challenges of mental health care in this country. A protocol for this systematic review was retrospectively registered with the Open Science Framework (OSF) registries on [27 October 2025], and is available at https://osf.io/ykrxn/overview (accessed on 27 October 2025).

### 2.1. Data Sources and Search Strategy

A team of librarians at the University of Gävle library performed electronic database searches in the English language, in consultation with the authors. The following databases were used: Medline (Ebsco), Cinahl (Ebsco), APA PsycINFO (Ebsco), Scopus (Elsevier), Web of Science (Clarivate), and Cochrane Library. The formal search was limited to publications written in the English language and published between 2012 and 2023.

Based on initial test searches and gold standard articles provided by the authors, a range of free text and index terms were retrieved to develop comprehensive query strings. The terms were structured using Boolean search logic. According to this principle: (interest OR interest) AND (context OR context), the connected topics of interest (mental health care) and context (Iran) were covered. No additional citation searches were performed. The outcome of the formal search, after adding the automatic language and date limitations, resulted in a total of 9336 records (Medline *n* = 1713, Cinahl *n* = 1190, APA PsycINFO *n* = 604, Scopus *n* = 2958, Web of Science *n* = 1827 and Cochrane Library *n* = 1044). After duplications were removed, 4397 relevant citations were identified.

A database search specialist (one of the co-authors who lives in Iran) also performed database searches in consultation with the authors. The search was conducted in Persian academic databases, including SID, Magiran, and Noormags, according to the same search strategy described above. The outcome of the formal search, after adding date limitations, resulted in a total of 241 records (SID = 78, Magiran = 91, and Noormags = 72). After duplications were removed, 113 relevant citations were identified.

Gray literature was excluded due to concerns about a lack of peer review and potential variability in methodological quality.

### 2.2. Study Selection

The inclusion criteria for the search at the University of Gävle consisted of scientific, peer-reviewed empirical original studies published in English between 2012 and August 2023. Qualitative and quantitative full-text articles, commentaries, editorials, correspondences, and brief reports were included. The exclusion criteria consisted of studies that reported on molecular or genetic diagnosis, healthcare professionals’ experiences, case studies, studies on different kinds of therapy or counseling and their effectiveness, studies on cost effectiveness, stigma, and attitudes toward patients with MHD, studies related to COVID-19, AIDS, and studies on Iranian MHD as immigrants in other countries. Studies that were not published in the English language were excluded as well. The inclusion criteria for the search in Persian databases were as described above, except that the language was Persian, and the peer-reviewed empirical original studies published between 2012 and August 2023 were included in the first phase. All retrieved citations were imported into an EndNote X8 digital library.

**Figure 1:**
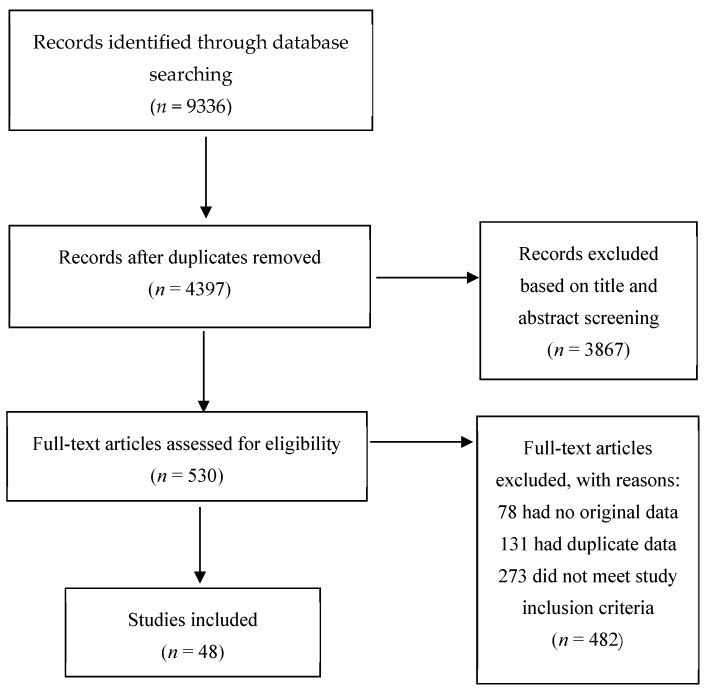
PRISMA Flowchart 1 (Articles from databases Medline, Cinahl, APA PsycINFO, Scopus, Web of Science, and Cochrane Library).

**Figure 2:**
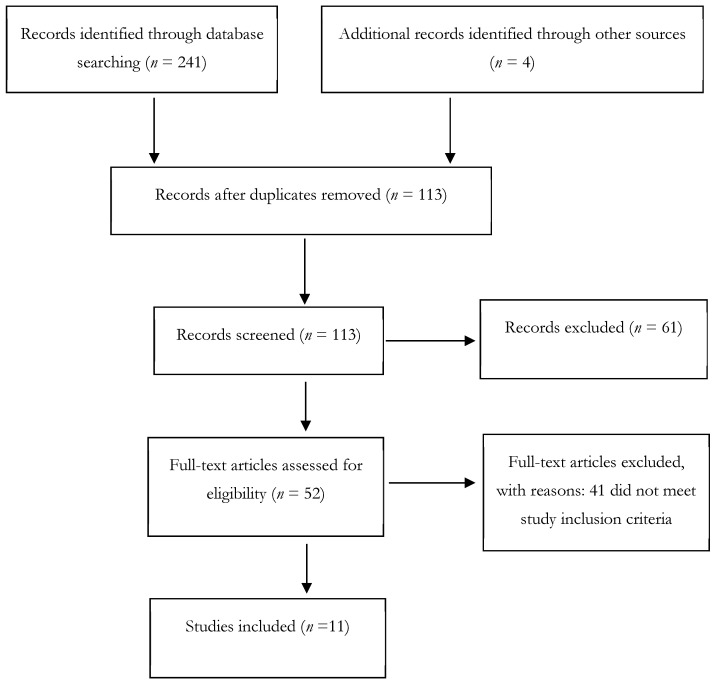
PRISMA Flowchart 2 (Articles from databases SID, Magiran, Noormags).

Following the predefined inclusion and exclusion criteria, the authors carefully reviewed the titles, keywords, and abstracts of all identified citations. Any citation that did not meet the eligibility criteria was excluded at this stage. Full-text versions of the remaining citations were then obtained, and these articles were examined in detail to reach a consensus on their final inclusion. Our screening process was conducted without blinding any bibliographic fields, as we determined that the practical challenges of this procedure outweighed its benefits for this specific review. Instead, we prioritized rigorous methodological safeguards to minimize bias, including dual-independent screening based on pre-defined criteria. For both the international and Iranian databases, the title/abstract and full-text screening were performed independently by two authors. To ensure independence, the screeners worked separately and without consultation during the initial assessment of all records. Their decisions were recorded independently. Any conflicts between the two screeners were resolved through a consensus discussion as a reconciliation procedure. It is worth noting that all screening of Persian-language records was conducted independently by native Persian-speaking members of our author team (SZ and FO). This ensured that the initial eligibility assessment was linguistically and culturally accurate. The study selection process is summarized in the PRISMA flow diagrams (Figure 1 and Figure 2).

As shown in Figure 1, the search of international databases resulted in the identification of 9336 records. After the removal of duplicates and screening, 48 studies were included for data extraction and synthesis.

As demonstrated in Figure 2, the search of Iranian databases and other sources resulted in the identification of 245 records. After the removal of duplicates and screening, 11 studies were included for data extraction and synthesis.

### 2.3. Data Extraction

Data extraction was conducted in two primary stages, both performed by humans. First, a training and calibration stage was held where all extractors practiced using the piloted data extraction form on a common subset of studies to ensure consistent understanding. This was followed by the main extraction stage, where two extractors worked independently and in parallel to extract data from all included studies into a standardized form. The extracted data were then compared, and any discrepancies were resolved through consensus discussion. The data were extracted based on study characteristics (study aims, sample, methods, outcomes). All the included articles were assessed using the Johns Hopkins Nursing Evidence-Based Practice Research tool. This quality guide is useful in estimating both the evidence level and quality of scientific studies. Each study was independently appraised by two reviewers and received both a quality rating (High, Good, Low) and an evidence level (I to V). Most included studies were rated as evidence levels II–III with a quality rating of B, indicating high to moderate methodological rigor, according to the Johns Hopkins tool. It should be noted that data from the included Persian-language studies were extracted by the same native speakers (SZ and FO). Key extracted data, including study characteristics and results, were then independently back-translated into English by a third bilingual researcher (SA) to verify conceptual accuracy and consistency.

### 2.4. Data Analysis

The Excel was used to organize and summarize the data by year, title, aim, study design, and findings [19]. Tables were used to organize extracted information. Given the anticipated heterogeneity in study designs and outcomes, we planned a narrative synthesis as our primary procedure. This was structured around a systematic textual summary, grouping findings by key themes or topics relevant to our research questions. The conclusions were drawn by interpreting the patterns and consistencies within the synthesized evidence. Inductive approach and thematic analysis (TA) were used to synthesize themes from the extracted data in order to respond to the research questions [20,21]. Using TA will contribute to approaching the text with certain research questions in mind, and to structuring data into patterns of meaning or interpretative themes in relation to research questions. TA is used because it provides a way to bring findings from studies that used different methodologies, populations, and contexts together and into coherent themes. Moreover, given the explanatory nature of our research question, TA provides the necessary flexibility to capture diverse perspectives across studies.

The synthesis was conducted through a collaborative process to ensure reliability. The analysis involved one of the authors (SA) by reading sources from English databases and two authors (SZ, FO) by reading resources in Persian from Iranian databases. Through each source, we could highlight data relevant to the research question. A preliminary analysis was written. The other authors (FA, RS) read through this preliminary analysis and discussed the relevance of each theme in relation to the research question, identifying possible areas of repetition or overlap before reaching a completed set of themes. This was followed by a critical review and discussion phase involving the entire research team, who challenged the relevance and distinctness of themes, and refined them through consensus.

## 3. Results

The complete search matrices for electronic database searches in the English language are available as Supplementary Material [see Additional File S1]. The included studies were 59 [see Additional File S2]. Four themes emerged from the included articles: *Prevalence of MHDs in Iran*, *Mental health organization*, *Inequality in mental health*, and *Challenges of Iranian mental health services*. Three of the themes were further subdivided into sub-themes.

### 3.1. Prevalence of MHDs in Iran

The prevalence of mental disorders in 2013 was estimated at approximately 20%, accounting for about 14% of the total national disease burden [22]. Based on the Iran Burden of Disease Study, mental disorders represent 10.25% of the country’s overall disease burden, while substance-related disorders add another 4.5%. The most common mental disorders include depression (26% of the mental disorder burden), addiction (24%), and bipolar disorder (12%). Overall, mental disorders rank second after cardiovascular diseases in terms of total burden. Additionally, depression among women contributes to a higher disease burden compared with other conditions [23].

In 2019, 6.7 million incident cases and 15.7 million prevalent cases of mental disorders were found in Iran. Data show that between 1990 and 2019, the number of DALYs due to mental disorders increased from 1.1 million to 2.05 and age-standardized DALY rates increased 1.8% [24]. In 2019, depressive disorders, anxiety disorders, and bipolar disorder were the leading mental health conditions contributing to the age-standardized DALY rate [25]. During the same year, depression, headaches, and anxiety disorders ranked among the ten most prevalent causes of death and disability [25]. Substance use has also emerged as a concern within Iranian high school populations [26].

A study examining the epidemiology of MHDs among Tehran residents from 2016 to 2019 reported that nearly 37% of the population experienced mental health issues, with a higher prevalence among women (45%) compared to men (28%). The highest rates of MHDs were observed in individuals aged 25–34 and those over 75 years. The most frequently reported disorders included depression (43%) and anxiety (40%), followed by somatization (30%) and social dysfunction (8%). MHDs were more frequent in the southeast regions (very low socioeconomic regions) of the city. The study concludes that the prevalence of MHDs among Tehran residents is considerably higher than national averages, with an estimated 2.7 million individuals in need of mental health care services [27].

#### 3.1.1. Suicide

In Iran, the suicide rates are reported at 5.3 per 100,000 people for both sexes, with 3.6 for females and 7.0 for males. The highest rates occur in the summer months, accounting for 35.2% of all suicides, which is approximately 13% higher than in other seasons. Suicide attempts are more prevalent in urban areas compared to rural regions, likely due to the increased stress associated with urban lifestyles. The most common methods of suicide in Iran include drug use, attributed to its accessibility, and self-immolation. The primary causes of suicide are family conflict (32%), marital problems (26%), economic constraints (12%), and educational failures (5%). Various complex factors contribute to the risk of suicidal behavior, including biological, psychological, familial, socioeconomic, political, and geographical influences [28]. A study conducted in Ilam province indicates that the risk of suicide is particularly increased among unemployed individuals [29].

#### 3.1.2. Care and Treatment

A significant number of Iranians with mental disorders, approximately 65.3%, did not receive psychiatric care according to the 2010 National Mental Health Survey (NMHS) [30]. Two-thirds of Iranians diagnosed with MHD do not receive mental health interventions because the mental health services have been inadequately distributed in Iran [31]. Mental disorders also place a significant financial burden on families in Iran. According to the NMHS, over 30% of a household’s annual income is allocated to psychiatric treatment for a single patient [30]. However, another study suggests that under the new socio-mental services model, these expenses are relatively modest in comparison with Iran’s GDP per capita [22].

MHDs among children and adolescents are also prevalent in Iran. The unmet need for mental health care in this age group is even greater than in adults, as child and adolescent mental health services remain considerably less developed than those available for adults [30,32]. There are no specialist psychological clinics for children and adolescents in the governmental sector in Iran.

### 3.2. Mental Health Organization

Mental health services in Iran were established in the 1940s within university hospitals. In the 1970s, community-oriented mental health care was first introduced by the Ministry of Health and Welfare [33]. The integration of mental health services into the public healthcare system began in 1986 through a model that incorporated mental health care into primary healthcare at the local level. Evidence from previous studies shows that this integration program enhanced health workers’ knowledge and improved their skills in patient screening [34]. The initial mental health program, piloted between 1992 and 1994, now serves approximately 18 million people (about 80%) in rural regions and 10 million (around 20%) in urban areas, primarily focusing on severe mental health conditions such as epilepsy and mental disability. However, it is insufficient for urban areas, particularly for disorders such as depression, anxiety, schizophrenia, bipolar disorder, and suicide attempts [35]. An evaluation of the mental health integration program in 2005 revealed that physicians involved in the program received inadequate supervision regarding mental health initiatives. Moreover, over 46% of essential mental health medications were unavailable in rural centers, and there was a lack of oversight of physicians’ activities in these rural facilities by the scientific advisor of the mental health program [35].

The integration program has since been revised. According to a 2016 study, the intended objectives across various domains, including service delivery, training, information systems, evaluation, advocacy, provision of essential medicines, quality improvement, and financial and administrative affairs, were redesigned. Specific objectives and measures were identified for each strategy [36].

According to the latest data available, there were 20 mental health workers per 100,000 Iranians in 2017 [37]. In 2013, the number of psychiatrists was about 1000, and the number of clinical psychologists was 174 [22].

The budget allocated for mental health amounts to about three percent of total health expenditure, which is relatively low given the significant contribution of MHDs to the national burden of diseases. A previous study estimates the mental health cost per capita to be 1.73 US dollars, while another study suggests that the cost could be as high as 16.4 US dollars per capita [23]. Notably, 18% of the total expenditure on mental health is directed towards mental hospitals [38]. Approximately 53% of the population has access to essential psychotherapeutic medicines free of charge, with a coverage of at least 80%. All mental disorders are covered under the social insurance schemes, with certain limitations on the duration of hospital stays [38]. The incorporation of mental health services into the primary health network represents a significant stride towards the progress and advancement of mental health care services in the country. Despite these strides, the detection rates of mental disorders remain low, and the system falls short of optimal performance, particularly in urban areas where the primary health care (PHC) network is less efficient compared to rural areas and a robust private sector offers patients direct access to specialists and subspecialists. Moreover, there is no established framework for the integration of child and adolescent mental health care into the PHC system, which raises uncertainty regarding the adequacy of mental health care provided to children and adolescents within the system [39].

In 2012, Iran introduced a new Mental Health Promotion Program [35]. This initiative proposed three key strategies for achieving its objectives: 1. Improving the quality and quantity of mental health services; 2. Enhancing mental health literacy among diverse social groups; 3. Investing in the reduction of mental health risk factors.

Implementing these strategies necessitates a re-examination of the existing mental health care system. A pilot study, known as SERAJ, proposed in 2021 by stakeholders and researchers, considered both preventive and treatment measures for MHDs. This study proposes that all citizens should have access to primary and secondary mental health services [31]. Additionally, it was suggested that strategies should focus on providing integrated care to individuals with mental health and substance use disorders, given the increasing and unmet burden of these conditions [25].

#### 3.2.1. Chain of Providing Mental Health Care Within Iran’s Primary Health Care

**Figure 3:**
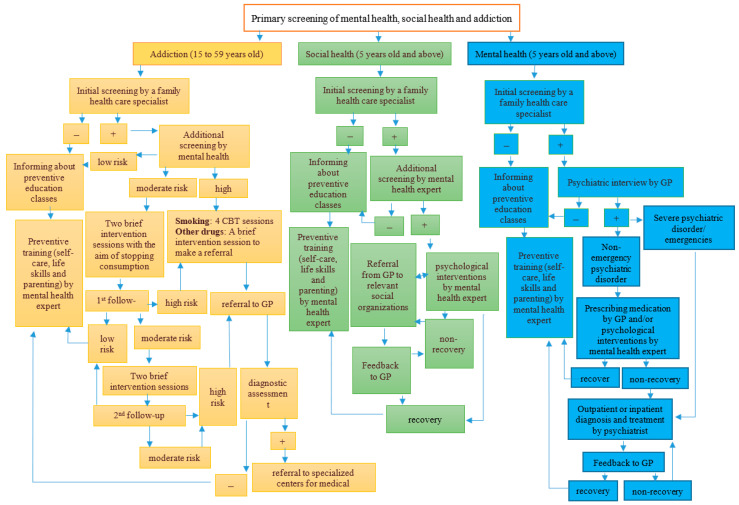
Flowchart of mental health services in Iran’s primary health care system [41,42].

Figure 3 illustrates the process of providing mental health services within Iran’s primary health care. Individuals visiting primary health care centers are assessed using three distinct forms for psychological health, social health, and addiction when creating or periodically updating their health files. The procedure for accessing and utilizing primary health services is uniform for all individuals, including both Iranian citizens and documented immigrants (mostly of Afghan origin), and is systematically recorded. Primary health care and referrals to specialized levels within Iran’s health system are managed by the public sector. Although approximately 75% of outpatient mental health services in Iran are provided by private clinics, psychologists, and psychiatrists [40], the private hospitals and private counseling and psychotherapy centers operate independently of this process and do not have a defined role or systematic procedure within the public sector referral system. Mental health services are available for individuals aged five years and older. If children under five exhibit clinical symptoms, they are examined by a general physician for developmental issues. Based on the physician’s diagnosis, they may be referred to a pediatrician at a hospital. No specific psychological services are provided to these children at health centers, although their parents can access parenting training services. All mental health services within Iran’s primary health care system are free of charge. However, if a general practitioner refers a patient to a hospital-based psychiatrist, the patient is required to pay a fee as determined by the Ministry of Health [41,42].

Regarding the Mental Health pathway, it should be noted that “emergency cases” include individuals with suicidal thoughts and attempts, acute anxiety symptoms (such as panic attacks), acute psychosis symptoms, and aggression. The Social Health pathway targets two groups: domestic violence (spousal abuse and child abuse) and vulnerable families (individuals or family members with physical or mental disabilities, chronic psychiatric illnesses, recent divorce or death of a spouse, unemployment, addiction, and imprisonment). Domestic violence in the form of spousal abuse includes married women aged 18 to 59. Given that a high percentage of reported violence is against women, psychological services are provided only to women experiencing violence. No specific services have been considered for violence against men or the elderly. The program to prevent violence against children includes those aged 5 to 18. In the Addiction pathway, services are provided to individuals aged 15 to 59. Those under 15 who are involved in addiction issues are referred directly to the center’s general practitioner [41,42].

#### 3.2.2. Private Sector

One of the unmet needs in Iran’s mental health system is the integration of the private sector into primary health care. The rapid urbanization of Iranian society in recent decades has made community-oriented health care less feasible in urban areas, as the private sector, which is not typically community-focused, dominates these areas. The majority of outpatient mental health services in Iran are provided by the private sector, while the public sector predominates inpatient services [40]. The lack of a clearly defined public–private partnership mechanism may contribute to the underdiagnosis and undertreatment of psychological disorders [43]. A model proposed in 2017 emphasizes the importance of considering the capacity of the private and non-profit sectors in providing both basic- and advanced-level mental health care services for socio-mental issues [35].

### 3.3. Inequality in Mental Health

#### 3.3.1. Socio-Economic Status (SES)

The data suggest a strong link between SES and mental health in Iran. Lower SES is associated with higher rates of mental disorders, aligning with the theories of social determinants for health. It means that poverty-related stressors can lead to mental health issues and restrict access to quality care [44]. One study emphasizes the critical role of social conditions and supports global evidence indicating that social determinants play a more substantial role in health and disease than individual or psychological factors [45]. Another study revealed that individuals who perceived their social status as low were more likely to report poorer overall responsiveness [46]. Furthermore, another research has demonstrated that the risk of mental disorders decreases with increasing educational level [47]. Substance-related psychiatric disorders are a significant problem in Iran. Evidence suggests that most individuals with these disorders are young, male, unemployed, with lower SES and lower education, living in urban areas [48,49]. Despite the considerable burden of mental disorders, the use of psychiatric services in Iran remains limited, largely because of financial barriers and inadequate insurance coverage. This limits access for low-SES households, who often cannot afford these services, while high-SES households have greater access due to their financial resources [50,51]. The substantial expense of mental health services can pose a major obstacle for patients with mental disorders, particularly those with low SES, in accessing care [52,53,54]. A study indicates that 25.8% of households with severe mental disorder patients experienced Catastrophic Health Expenditure (CHE), with variables such as age and education level of household heads being significant predictors of CHE [55].

#### 3.3.2. Gender

Gender disparities are evident in the mental health landscape of Iran. Women are at a higher risk of mental disorders and attempted suicides compared to men. Gender norms and socio-economic inequities may lead to MHDs among women, showcasing the layered impact of gender and economic status on mental health.

The prevalence of mental disorders among Iranian women is relatively higher, as evidenced by a relative risk of 1.6 compared to Iranian men [30]. There is a significant relationship between perceived gender inequality and mental health disorders among Iranian women [55]. A study conducted in Ilam province confirms that females have a greater tendency for attempted suicide, while completed suicide rates are higher among males [29]. The intersection of being a woman, having low SES, and residing in deprived provinces results in a higher incidence of chronic diseases and mental health issues among women. This is primarily due to the financial deprivations and socioeconomic inequities, such as inadequate nutrition, poverty, poor housing, and low income [56].

The topic of gender differences in the prevalence of psychiatric disorders has been a matter of much discussion. In Iran, the adoption of Bipolar Spectrum Disorder (BSD) as a diagnostic construct and the concept of ”soft bipolarity“ are related to a considerable gender disparity in rates of diagnosis. A qualitative study posits that the high rate of diagnosis of BSD among patients in Iran is influenced by various factors, including gender, sociocultural, political, and economic factors, as well as the diagnostic practices of biomedical psychiatry [57].

#### 3.3.3. Belonging to Ethnic Minorities or Migrant Population

The Persian ethnicity represents the majority of Iran’s population, but nearly a dozen other ethnicities account for over a third of the nation’s 89 million inhabitants. However, the underdevelopment of many minority-populated provinces has resulted in inadequate healthcare services for these communities. Regions such as Ilam, Khuzestan, Kurdistan, Lorestan, and Sistan-Baluchestan, which are home to minority populations, remain underdeveloped, leading to higher poverty levels and poorer health outcomes [58].

The prevalence of mental disorders varies considerably across different provinces in Iran, ranging from 3.6% to 62.6% [59]. A significant portion of the urban population in Ilam is estimated to suffer from mental disorders [60], and it is believed that at least 26.1% of the population in this region is affected by one or more mental disorders [61]. Kermanshah, Chaharmahal and Bakhtiari, and Kohkilouyeh and Bouyerahmad are also identified as deprived provinces in Iran with high proportions of MHDs, especially among people with low SES and children and adolescents [62,63,64].

Iran, like many other countries in the world, is a host for immigrants from different countries. Currently, there are approximately three million Afghan immigrants living in Iran, of which only one million are documented [65]. A study conducted in an Iranian city with a high concentration of Afghan immigrants found that the rate of psychiatric disorders among these immigrants was 34.5%, with a disproportionately high rate among women. The study also revealed that the rate of psychological problems within this population was approximately twice that of the host society, and identified various non-cultural factors that contributed to these disorders, including lower socioeconomic status, stressful job conditions, type of residence (centralized or decentralized), and worries about changes in Iran’s immigration policies [66,67].

#### 3.3.4. Age

##### Children and Youth

Mental health issues among Iranian youth are influenced by various intersecting factors, including gender, SES, and ethnicity. Adolescents—particularly girls, those from low-SES backgrounds, ethnic minorities, and Afghan immigrants—are at higher risk of anxiety, depression, and suicidal behavior. The intersection of these factors creates a complex web of influences that significantly affects young people’s mental health, highlighting the need for targeted interventions that address these intersecting vulnerabilities.

A significant proportion of Iranian children and adolescents suffer from mental and behavioral health issues, with estimates ranging from 16.7% to 36.4% [68,69].

There is a complex interplay between adolescent anxiety and depression, age, gender (with secondary school girls being at higher risk), SES factors like parental unemployment, and belonging to ethnic minorities [70,71,72,73,74]. Moreover, a study from a deprived province (Ilam) indicates that the suicide rate is highest among the 15–24 age group [29]. Another study revealed that 28.7% of youth in deprived provinces in western Iran suffer from psychiatric disorders [75].

##### Older Adults

Older adults in Iran face significant mental health challenges, with depression being highly prevalent. Socio-demographic factors like gender, education, and income are closely linked to mental health outcomes in this group. The increasing rate of suicides among older adults, particularly in southern Iran, highlights the intersection of aging, socio-economic conditions, ethnicity, region of residence, and mental health. This demographic shift necessitates tailored mental health services that address the specific needs of older adults.

As a developing nation, Iran has undergone notable demographic changes over the past four decades, shaped by shifts in health policies. By 2050, it is projected that 25% of Iran’s population will be older adults (60+). Depression is prevalent among Iranian older adults, with women experiencing higher rates of depression than men. A study found that 43% of Iranian older adults were suffering from depression, with no significant difference based on gender or marital status [76]. Other studies support the correlation between socio-demographic factors and mental health among Iranian older adults. A study in Zanjan city found significant associations between gender, education, occupation, and income with mental health among older adults [77]. Moreover, a study discovered that sociodemographic factors, health conditions, and living arrangements were contributory factors to the development of depression among older adults, with the nature of these associations influenced by the economic class [78].

In a study conducted in a southern province of Iran, the epidemiology of suicide among older adults (aged 65 and above) between 2011 and 2016 was analyzed. The findings revealed that the rates of attempted suicide and completed suicide were 21.47 and 4.52 per 100,000 people, respectively. The study also highlighted an alarming upward trend in both attempted and completed suicides over the study period [79].

#### 3.3.5. Sexual Orientation and Gender Identity

The mental health needs of Iranian LGBTQIA+ individuals, particularly those who are gay or lesbian, are inadequately addressed. Homosexuality and all other types of sexual orientation except heterosexuality are forbidden according to the law of the Islamic Republic of Iran. To obtain legal permission for sex reassignment surgery, individuals must present medical proof of gender identity disorder, which is assessed by psychiatrists and clinical psychologists in authorized clinics [80]. A study on transgender people’s mental health services revealed that a lack of access to healthcare tailored to their needs is a significant challenge. Many transgender individuals refuse treatment for issues related to their gender and body discomfort, often due to stigma, prejudice, invasive questioning, and restrictive attitudes of healthcare staff [81].

### 3.4. Challenges of Iranian Mental Health Services

Promoting mental health and preventing MHDs are key concerns for every country, requiring effective structure and management of mental health care. Identifying the challenges that contribute to deficiencies and shortages is necessary to achieve these goals.

#### 3.4.1. Inadequate Mental Health Care Policy, Plans and Programs

Previous studies in Iran have identified multiple challenges facing mental health services, including low awareness and sensitivity among top policymakers within the Ministry of Health and universities, inadequate prevention programs—particularly regarding social determinants of health—limited intra-sectoral coordination and inter-sectoral collaboration [82,83], insufficient mental health programs in terms of coverage and intensity, a lack of adequate services in urban areas, and weaknesses in the structure, processes, and resources (including human resources, information systems, and budgets) of the mental health system [22,84]. Effective mental health enhancement programs in Iran require accurate measurement of the current mental health status and the selection of key indicators. One study has proposed essential indicators such as the annual prevalence of mental disorders, suicide rates, the number of mental health professionals, and mental health expenditures, which can be used to monitor progress in policy reforms and community mental health services [85]. Additionally, delivering optimal nursing care in psychiatric wards necessitates skilled psychiatric nurses, a stable and accountable organizational structure, community-based care, anti-stigma initiatives, and improved administrative processes. Fidelity to protocols and documentation, as well as continuous evaluation and quality improvement, are also essential [86].

#### 3.4.2. Impact of Urbanization on Mental Health Care

Rapid urbanization is another challenge that has been identified by previous research as having a significant impact on mental health and psychiatric care in Iran. With over two-thirds of the population living in urban areas, rapid urbanization has profound implications for mental health and access to psychiatric care [87]. To achieve a comprehensive model of urban mental health services, providing patients with services ranging from prevention and care to treatment and rehabilitation, there is a need for inter-organizational collaboration [22,88]. The Ministry of Health and Medical Education (MOHME) should primarily concentrate on enhancing mental health services in urban and peri-urban areas, improving mental health literacy across diverse populations, and mitigating risk factors associated with mental health issues [22].

#### 3.4.3. Poor Responsiveness Dimensions

In 2000, the World Health Organization (WHO) identified health system responsiveness as a core objective of health systems. WHO’s concept of responsiveness can be applied to mental health services in Iran, aiding policymakers in improving these services [46]. A study of 500 public mental health service users in Tehran found that 47% of participants reported low responsiveness. Among the different domains of health system responsiveness, confidentiality and dignity scored the highest, whereas autonomy, access to care, and quality of basic amenities were the lowest performing. Participants with lower social status were more likely to report poor overall responsiveness. Although attention and access to care were rated poorly, participants considered them highly important. The findings indicate that assessing responsiveness can inform the development of more patient-centered mental health care systems, ensuring greater respect for patients [89].

#### 3.4.4. Poor Training in Mental Health Care for GPs

Previous research indicates that Iran possesses well-established primary care networks staffed by general practitioners (GPs) who deliver healthcare services across all age groups. To enhance the provision of mental health services, collaborative care networks have been established to strengthen GPs’ capacity to address mental health issues in adults, children, and adolescents [35,90]. According to a study, GPs reported encountering a wide range of emotional and behavioral problems in children. They also highlighted the need for further training in diagnosis and management, especially in skills related to interviewing and communicating with children. Additionally, general practitioners emphasized the importance of understanding the legal aspects of treating children, including cases involving potential child abuse [90].

#### 3.4.5. Deep Gaps in Mental Health Care Services

A study [91] suggests that Iranian policymakers should prioritize the main gaps in public mental health care services, including increasing access, continuity of care, coordination of service delivery, and comprehensiveness of care. The study also emphasized the need for a reform that integrates mental health services into PHC, noting that the necessary infrastructure, including human and financial resources, and support from senior authorities within the MOHME, are essential for ensuring the continuity and enhancement of services. To ensure the changes in the public mental healthcare system are successful and enduring, it is recommended to monitor and evaluate the service model, as well as to maintain sustainable financial resources [91].

#### 3.4.6. Low National Budget for Mental Health Care

A study analyzed the performance of Iran’s Community-based Mental Health Centers (CMHCs) and found challenges in the program’s implementation. These challenges included the difficulty in attracting more general physicians to the CMHCs, as well as the influence of local culture and attitudes on the effectiveness of the program [92]. Another study estimated the impact of expanding a new mental health model (from basic mental health care in primary healthcare to community-based mental health centers) at the national level, as well as its associated costs. The study projected that the expansion of this model would lead to a total of 1,702,755 healthy life years gained between 2020 and 2030. Assuming a base case scenario cost of 1,363,581,654 US dollars, each healthy life year gained would cost approximately 801 US dollars. Based on WHO criteria, values ranging from 724 to 1119 US dollars across eight different scenarios were deemed cost-effective, considering Iran’s 2018 GDP per capita of 5550 US dollars. Mental health expenditure in Iran accounts for approximately 3% of total health spending, with a per capita cost of 1.73 US dollars, which is relatively low given the share of mental, neurological, and substance use (MNS) disorders in the national disease burden. The study indicated that increasing the per capita investment to 16.4 US dollars for scaling up a comprehensive mental health service model could persuade high-level policymakers to allocate a larger portion of the health budget to mental health [22].

## 4. Discussion

This systematic review aimed to describe the status of mental health and mental health care in Iran between 2012 and 2023, addressing inequalities and mapping existing challenges in this sector. The results are discussed in light of the social determinants of health [15] and the social mechanism model [16]. These frameworks outline four primary mechanisms through which health inequities arise among social groups: social stratification, differential exposure, differential vulnerability, and differential consequences. Using these mechanisms, potential policies to improve mental health care in Iran are explored.

### 4.1. Social Stratification

In the Diderichsen et al. model, the foundational and most influential social mechanism is social stratification [16]. Societies organize individuals into hierarchical structures based on attributes linked to their social position and context. Social context includes features of the broader society or culture, such as community resources, social norms, and communal expectations [16]. It also encompasses societal factors that influence the distribution of power, wealth, and risks, including access to healthcare services and opportunities for workforce participation. In Iran, the social context is hierarchical due to the organization of its political and cultural systems. Social stratification in Iran results in a significant divide between different social classes [45,93]. In 2016, studies found that unemployment, the Gini coefficient, and inflation were found to be significantly negatively associated with psychological health. Both unemployment and inflation rates, as well as income inequality, have a significant impact on suicide rates among men and women [94,95]. Iran’s 2022 Gini coefficient of 40.9 [96] and the Human Development Index (HDI) of 0.774 [97] highlight significant social inequalities in Iran.

Social stratification in Iran is also influenced by gender. In 2021, the HDI value for females in Iran was 0.704, compared to 0.800 for males, resulting in a Gender Development Index (GDI) value of 0.880, placing Iran in Group 5 and below the world average [98]. This gender disparity contributes to the inequality and stratification within Iranian society.

Age is another factor of social stratification in Iran, with officials warning about the potential emergence of a demographic ‘tsunami’. It is projected that Iran could have one of the five largest older adult populations by 2050, with nearly 11% of Iranians currently over 60 years old. This figure is expected to significantly increase in the coming years [99]. The needs of Iran’s older adult population are not sufficiently met, leading to some demonstrations among retired older adults for better pension wages. On the other hand, over 60% of Iran’s population is under 30 years old, facing issues such as unemployment, economic barriers to family formation, and limited representation in macro-level decision-making processes [100,101].

Belonging to an ethnic minority group or being an immigrant is another factor of social stratification in Iran. Ethnic groups residing in minority-populated regions, including Khuzestan, Kurdistan, and Sistan-Baluchestan, continue to experience underdevelopment, higher poverty rates, and poorer health outcomes [58]. Afghan refugees in Iran have also reported experiencing social discrimination [102].

### 4.2. Differential Exposure

Social stratification is at the heart of understanding health inequities across social groups, as individuals’ socioeconomic status and social positions can lead to different types of risk exposure. The second mechanism connecting social position to health outcomes is differential exposure to health risks, indicating that increased exposure to risk factors raises the probability of adverse health outcomes throughout life [103]. In Iran, this is reflected in the high prevalence of mental health disorders among children and adolescents [68,69,70,71,72,73,74,75]. An Iranian inquiry investigating the social determinants of mental health identified several risk factors contributing to impaired mental health, including unemployment, income insecurity, and increased living costs, which can collectively lead to a decline in social welfare [51].

### 4.3. Differential Vulnerability

The Whitehall studies have shown that the interplay between social context and the distribution of resources is a key determinant of health outcomes across socioeconomic groups, with poorer health outcomes observed in groups with fewer resources and opportunities [104]. Accumulated health risks within social groups contribute to the third mechanism influencing health: differential vulnerability, where some groups are more susceptible even when risk factors are evenly distributed, as the effects of these risks vary between groups. In resource-limited societies, privileged groups tend to disproportionately access high-value resources, such as quality housing and healthcare, enhancing their resilience to health risks. Drug use in Iran offers a good example of how differential vulnerabilities manifest in society [23,105]. Drug users who develop MHDs and/or die by suicide tend to be young males from low SES [29]. Although privileged groups in Iranian society may also use drugs, they are less vulnerable to the risk of MHDs due to their greater access to resources that can minimize harm [29].

### 4.4. Differential Consequences

Social stratification can lead to varying consequences as health risks accumulate over time, affecting both individuals and communities. In Iran, where a strong safety net for mental healthcare is lacking [50,51,52,53,54], privileged populations possess greater resources to cover medical costs and compensate for lost productive hours. Conversely, individuals from lower socioeconomic backgrounds may face substantial losses in both time and income due to limited workforce participation, further exacerbating their disadvantage. Thus, the social repercussions of falling into mental health distress are disproportionately severe for disadvantaged groups [104].

The absence of power and material resources subjects disadvantaged groups to higher stress levels, which may lead individuals to adopt unhealthy coping strategies, such as drug or alcohol use, to alleviate this stress. As a result, those from disadvantaged backgrounds are more likely to engage in risky behaviors as a coping strategy, making them more vulnerable to the negative consequences associated with these risks [104].

### 4.5. Policy Points to Impact Mental Health Disparities

The mechanisms outlined above suggest potential policy interventions to disrupt these processes and enhance health outcomes. According to the model [16], reducing social stratification can be influenced by broader macro-level policies aimed at redistributing wealth and power. In the case of Iran, prioritizing universal mental healthcare should be a key focus at the macro level. Additionally, implementing appropriate measures to expand basic and supplemental insurance coverage for individuals with MHD should also be prioritized at this level, as it can help alleviate the financial burden on affected individuals and their families. WHO introduced necessary interventions for mental health promotion in the following levels: “(1) Primary prevention (preventing incidence of mental disorders); (2) Secondary prevention (early recognition and timely treatment of mental disorders); (3) Tertiary prevention (rehabilitation of the mentally ill patients to reduce the occurrence of any defect or disability)”.

#### 4.5.1. Improving Mental Health Care Policy and Programs and Closing the Gaps

Previous research from Iran highlights several key challenges facing mental health services, including low sensitivity among policymakers, inadequate prevention programs, poor intra-sectoral coordination, and insufficient inter-sectoral cooperation [82]. Additionally, there is a lack of inter-professional collaboration [106], inadequate mental health programs and services in urban areas, and deficiencies in the structure, processes, and resources of the mental health system [22,84]. The World Health Organization Assessment Instrument for Mental Health Systems (WHO-AIMS) was previously utilized [38] in 2006 to identify both areas of success and areas in need of improvement. To effectively address the existing mental health gaps in Iran, it is essential to update these assessments in light of demographic changes and evolving needs to enhance the provision of mental health services.

There is a pressing need to rapidly adapt and evolve mental health priorities and policies in Iran to align with the demographic landscape. Revising existing programs based on recent research findings in epidemiology, community needs assessments, cost-effectiveness analyses, and consideration of cultural and social factors is crucial [107].

#### 4.5.2. Increasing the National Budget for Mental Health and Taking Action to Address Pressing Needs

Increasing financial resources, enhancing management capabilities, and educating more psychologists and psychotherapists could significantly improve mental healthcare in Iran. Establishing dedicated mental health services for children and youth is another essential intervention. Addressing these fundamental needs, including ensuring equal access to mental health services, is crucial as they impact multiple health outcomes. Therefore, policymakers and providers should take action to address these pressing needs.

To reduce disparities in mental healthcare in Iran, providers should aim to distribute clinical services based on need, prioritizing treatment for the most severely ill. Developing mental health services in urban areas [35] and underserved provinces [58,59] requires the selection of specific mental health indicators [85] and continuous measurement of responsiveness to facilitate the advancement of mental healthcare systems [46]. Regional psychiatric surveys are essential, as significant differences exist among various regions regarding subcultural, socioeconomic, etiological, religious, and age factors. Increasing public awareness, establishing counseling centers, and providing culturally diverse training for counselors are critical steps. Policymakers in the provinces should also focus on creating platforms that foster social capital and improve infrastructure to enhance mental health services [32,50,60,108].

The most effective way to reduce exposures and vulnerabilities, thereby diminishing health disparities, is to promote policies that narrow the gap between social groups. From the perspective of social determinants of health, if providers are to influence these determinants, they should strive to minimize the accumulation of socially patterned disadvantages, which will help foster better and more equitable population health outcomes while lowering costs. Another consequence of social stratification is the categorization of individuals into different health insurance types. In Iran, implementing measures to enhance both basic and supplemental insurance coverage for patients with MHD and increasing the budget for mental healthcare [3,22] are fundamental steps toward achieving these goals. By addressing key risk exposures, a domino effect can positively influence the Iranian patients suffering from MHD.

#### 4.5.3. Expanding the Role and the Resources of Primary Care and Health Centers

To reduce social stratification in Iran, it is essential to continue integrating mental health services into public health programs and primary healthcare to enhance the utilization of psychiatric services [32,50]. Expanding the role of primary care in the timely detection, early intervention, and management of mental health issues among children and adolescents is particularly important for improving care for these populations [68,69,73,90], as well as for older adults [76]. This approach should be guided by a life course perspective, focusing on the most vulnerable populations during the earliest stages of life to prevent negative cumulative effects throughout their lifespan.

As the chain and challenges for mental health care show, the lack of trained staff and exhausted providers is a significant limitation for mental health care in Iran. According to existing policies, a clinical psychologist under the job title of ‘mental health expert’ is employed in each health center for every 40,000 people in a region [109]. It is expected that about 12% of individuals who undergo initial screening will test positive. All these cases are referred to the center’s general practitioner (GP) to complete the psychiatric form, and it is anticipated that about 50% will be diagnosed by the GP. This volume of patients imposes a heavy workload on the GPs. There is no specific system for making appointments with a mental health expert following a positive screening and referral [110]. In general, the challenges associated with providing mental health services within the primary health care system can be divided into two categories: internal challenges within the center and external challenges outside the center.

Regarding the internal challenges, it is worth noting that there are deficiencies at each stage of identification and referral among the health team (nurse, GP, and mental health expert), which affect the correct identification, referral, and use of mental health services. People in need of services may not be properly identified for several reasons, including the low level of healthcare providers’ expertise in primary triage, lack of a safe and private space for providing information, or the referrer’s reluctance to cooperate. Consequently, the likelihood of false negatives may increase. Another issue in the physician’s office is that mental health services may fail for patients due to various reasons, including physicians’ lack of training in identifying individuals with a mental health diagnosis [110]. Some physicians are reluctant to diagnose and prescribe treatments for mental health problems, often due to the overwhelming volume of their workload. In the third stage, when referring patients to a psychologist, the main issue is the lengthy process involved. A positive recommendation from both the healthcare provider and the doctor is necessary for a referral to a psychologist. This prolonged journey can discourage some individuals from continuing the process. Further, for various reasons, the referred person may be unwilling to cooperate [110].

In terms of external challenges, it is worth mentioning that some positive cases, which cannot be managed at the health center (level 1) and require specialized treatment, are referred to external specialized centers (level 2). In these cases, there is no specific link or communication between level 1 and level 2. Sometimes, it is not possible to secure a specialist appointment for a referred patient, which prolongs the process of receiving services and prevents proper follow-up. In recent years, the lack of communication between level 1 and level 2 has been partially addressed with the establishment of a limited number of SERAJ centers. However, these centers are not widespread and do not fully meet the needs of patients [110,111].

Further research is required to better understand the reasons behind the increasing prevalence of mental health disorders in Iran, particularly among vulnerable populations. Additionally, it is essential to identify and map the main obstacles to improving mental health care. What are the factors that hinder progress? Finally, the relationship between the private and public sectors should be further clarified, as the private sector bears a significant burden in the treatment and care of patients with mental health disorders.

### 4.6. Strengths and Limitations of This Study

The studies identified through the systematic search demonstrate a range of methodological strengths and limitations that could have affected the reported findings. A key strength of this review is its inclusion of a diverse set of studies in both English and Farsi, sourced from international and Iranian databases.

To our knowledge, this is the first review of its kind to explore the status of mental health and mental health care in Iran from an inequality perspective, aiming to address the existing challenges in this field. Findings from the cross-sectional studies included in this review highlight the potential importance of considering inequality in mental health. However, we acknowledge that further studies are needed to provide a stronger level of evidence. We also emphasize the importance of future studies that examine the obstacles to incorporating equity considerations into mental health policies and service delivery.

A limitation of our study is that we did not conduct a meta-analysis, as many of the included studies were heterogeneous in design and objectives; therefore, we cannot comment on the quality of data analysis or reporting in the reviewed papers. There is substantial gray literature on mental health in Iran that is not captured in this study. We chose to follow the PRISMA methodology and restrict our focus to peer-reviewed literature for consistency. Thus, there may be a potential risk of bias due to the exclusion of gray literature.

## 5. Conclusions

Analyzing the mental health landscape in Iran by addressing social inequalities reveals the intricate ways in which SES, gender, ethnicity, age, and sexual orientation influence mental health outcomes. This approach highlights the need for multi-faceted and inclusive strategies in addressing mental health challenges, ensuring that all affected groups receive appropriate and effective care. By understanding and addressing these social inequality factors, Iran can develop more robust and equitable mental health services.

The overarching challenges in Iran’s mental health care include inadequate sensitivity among policymakers, insufficient prevention programs, and poor coordination between sectors. These systemic problems are compounded by the socio-economic and demographic factors discussed, resulting in a fragmented and under-resourced mental health system. Addressing these challenges requires integrating equity perspectives into mental health policies and practices to ensure more equitable and effective care.

To improve mental health care in Iran, the following points are recommended:

- There is a need to evaluate and update the patterns for providing equal access to mental health services, considering demographic changes in Iran.
- Appropriate measures should be taken to increase both basic and supplemental insurance coverage for patients with mental health disorders, particularly in the private sector, which offers patients direct access to specialists and subspecialists.
- Increasing financial resources, enhancing management capabilities, and educating more psychologists and psychotherapists could improve mental healthcare in Iran.
- Establishing dedicated mental health services for children is an essential intervention.

## Data Availability

No new data were created or analyzed in this study.

## Supplementary Materials

The following supporting information can be downloaded at: https://www.mdpi.com/article/10.3390/healthcare13233131/s1, Additional File S1: Search matrices; Additional File S2: Peer-reviewed journal articles used in the Results section.
