# Supplementary material for: Mental Health and Mental Health Care in Iran: Addressing Social Inequalities
Source: Healthcare (Basel). 2025 Dec 1;13(23):3131. doi: 10.3390/healthcare13233131 (PMC12692221; doi:10.3390/healthcare13233131)
Supplement: Supplementary file 1 [file healthcare-13-03131-s001.zip › healthcare-3907829-supplementary/Additional file S1.pdf]

Search matrices

APA PsycInfo 230814

S1	TI (iran OR iranian OR kurdistan OR kurdish OR balochistan OR baluchistan OR baluchestan OR lorestan OR luristan OR lurestan OR loristan) OR AB (iran OR iranian OR kurdistan OR kurdish OR balochistan OR baluchistan OR baluchestan OR lorestan OR luristan OR lurestan OR loristan)	9,094
S2	DE "Mental Health Services" OR DE "College Mental Health Services" OR DE "Community Mental Health Services" OR DE "Mental Health Programs" OR DE "Psychological First Aid" OR DE "School Based Mental Health Services"	65,730
S3	DE "Psychiatry" OR DE "Addiction Psychiatry" OR DE "Adolescent Psychiatry" OR DE "Biological Psychiatry" OR DE "Child Psychiatry" OR DE "Community Psychiatry" OR DE "Consultation Liaison Psychiatry" OR DE "Forensic Psychiatry" OR DE "Geriatric Psychiatry" OR DE "Military Psychiatry" OR DE "Neuropsychiatry" OR DE "Orthopsychiatry" OR DE "Social Psychiatry" OR DE "Telepsychiatry" OR DE "Transcultural Psychiatry"	71,213
S4	DE "Psychotherapy"	76,006
S5	DE "Psychiatric Units"	2,373
S6	DE "Psychiatric Clinics"	1,853
S7	DE "Psychiatric Hospitals"	8,485
S8	DE "Community Mental Health Centers"	4,110
S9	TI ((mental OR socio-mental OR behavioral) N3 ("health care" OR "health services" OR "health center" OR "health hospital" OR "health facilit*" OR "health institution" OR "health system" OR "health plan" OR "health program*" OR "health policy" OR "health organization" OR "health organization" OR "health needs" OR "health insurance" OR "health disparities" OR "health ward" OR "health unit" OR "health clinic" OR "health department" OR "health model" OR "health strateg*")) OR AB ((mental OR socio-mental OR behavioral) N3 ("health care" OR "health services" OR "health center" OR "health hospital" OR "health facilit*" OR "health institution" OR "health system" OR "health plan" OR "health program*" OR "health policy" OR "health organization" OR "health organization" OR "health needs" OR "health insurance" OR "health disparities" OR "health ward" OR "health unit" OR "health clinic" OR "health department" OR "health model" OR "health strateg*"))	59,414
S10	TI (counseling OR counselling OR "behavioral therap*" OR "behavioural therap*" OR "cognitive behavioral therap*" OR "cognitive behavioural therap*" OR psychotherap* OR "psychodynamic therap*") OR AB (counseling OR counselling OR "behavioral therap*" OR "behavioural therap*" OR "cognitive behavioral therap*" OR "cognitive behavioural therap*" OR psychotherap* OR "psychodynamic therap*")	218,344
S11	TI (Psychiatr* N3 (Care OR Service OR Center OR Hospital OR Facilit* OR Institution OR System OR Plan OR Program* OR Policy OR Organization OR Organisation OR Needs OR Insurance OR Disparities OR Ward OR Unit OR Clinic OR Department OR Model OR Strateg*)) OR AB (Psychiatr* N3 (Care OR Service OR Center OR Hospital OR Facilit* OR Institution OR System OR Plan OR Program* OR Policy OR Organization OR Organisation OR Needs OR Insurance OR Disparities OR Ward OR Unit OR Clinic OR Department OR Model OR Strateg*))	63,535
S12	S2 OR S3 OR S4 OR S5 OR S6 OR S7 OR S8 OR S9 OR S10 OR S11	421,212
S13	S1 AND S12	700
	S13 AND filters activated: Publication year: 1990 – today, Language: English	604

CINAHL 230814

S1	MH "Iran"	32,633
S2	TI (iran OR iranian OR kurdistan OR kurdish OR balochistan OR baluchistan OR baluchestan OR lorestan OR luristan OR lurestan OR loristan) OR AB (iran OR iranian OR kurdistan OR kurdish OR balochistan OR baluchistan OR baluchestan OR lorestan OR luristan OR lurestan OR loristan)	26,334
S3	S1 OR S2	38,914
S4	MH "Mental Health Services+"	88,846
S5	MH "Psychiatry+"	18,426
S6	MH "Psychotherapy"	23,390
S7	MH "Psychiatric Nursing"	18,578
S8	MH "Psychiatric Service" OR MH "Psychiatric Units"	3,800

S9	MH "Hospitals, Psychiatric"	6,633
S10	TI ((mental OR socio-mental OR behavioral) N3 (“health care” OR “health services” OR “health center” OR “health hospital” OR “health facilit*” OR “health institution” OR “health system” OR “health plan” OR “health program*” OR “health policy” OR “health organization” OR “health organization” OR “health needs” OR “health insurance” OR “health disparities” OR “health ward” OR “health unit” OR “health clinic” OR “health department” OR “health model” OR “health strateg*”)) OR AB ((mental OR socio-mental OR behavioral) N3 (“health care” OR “health services” OR “health center” OR “health hospital” OR “health facilit*” OR “health institution” OR “health system” OR “health plan” OR “health program*” OR “health policy” OR “health organization” OR “health organization” OR “health needs” OR “health insurance” OR “health disparities” OR “health ward” OR “health unit” OR “health clinic” OR “health department” OR “health model” OR “health strateg*”))	33,806
S11	TI (counseling OR counselling OR "behavioral therap*" OR "behavioural therap*" OR "cognitive behavioral therap*" OR "cognitive behavioural therap*" OR psychotherap* OR “psychodynamic therap*”) OR AB (counseling OR counselling OR "behavioral therap*" OR "behavioural therap*" OR "cognitive behavioral therap*" OR "cognitive behavioural therap*" OR psychotherap* OR “psychodynamic therap*”)	82,257
S12	TI (Psychiatr* N3 (Care OR Service OR Center OR Hospital OR Facilit* OR Institution OR System OR Plan OR Program* OR Policy OR Organization OR Organisation OR Needs OR Insurance OR Disparities OR Ward OR Unit OR Clinic OR Department OR Model OR Strateg*)) OR AB (Psychiatr* N3 (Care OR Service OR Center OR Hospital OR Facilit* OR Institution OR System OR Plan OR Program* OR Policy OR Organization OR Organisation OR Needs OR Insurance OR Disparities OR Ward OR Unit OR Clinic OR Department OR Model OR Strateg*))	22,985
S13	S4 OR S5 OR S6 OR S7 OR S8 OR S9 OR S10 OR S11 OR S12	224,004
S14	S3 AND S13	1,454
	S14 AND filters activated: Publication year: 1990 – today, Language: English	1,190

Cochrane Library 230814

S1	MeSH descriptor: [Iran] explode all trees	2,802
S2	(iran OR iranian OR kurdistan OR kurdish OR balochistan OR baluchistan OR baluchestan OR lorestan OR luristan OR lurestan OR loristan):ti,ab,kw	12,163
S3	S1 OR S2	12,163
S4	MeSH descriptor: [Mental Health Services] explode all trees	9,316
S5	MeSH descriptor: [Psychiatry] explode all trees	1,218
S6	MeSH descriptor: [Psychotherapy] this term only	3,907
S7	MeSH descriptor: [Psychiatric Nursing] this term only	240
S8	MeSH descriptor: [Psychiatric Department, Hospital] this term only	112
S9	MeSH descriptor: [Hospitals, Psychiatric] this term only	287
S10	MeSH descriptor: [Community Mental Health Centers] explode all trees	142
S11	(((mental OR socio-mental OR behavioral) NEAR/3 (“health care” OR “health services” OR “health center” OR “health hospital” OR health NEXT facilit* OR “health institution” OR “health system” OR “health plan” OR health NEXT program* OR “health policy” OR “health organization” OR “health organization” OR “health needs” OR “health insurance” OR “health disparities” OR “health ward” OR “health unit” OR “health clinic” OR “health department” OR “health model” OR health NEXT strateg*))):ti,ab,kw	6,622
S12	(counseling OR counselling OR behavioral NEXT therap* OR behavioural NEXT therap* OR cognitive NEXT behavioral NEXT therap* OR cognitive NEXT behavioural NEXT therap* OR psychotherap* OR psychodynamic NEXT therap*):ti,ab,kw	57,246
S13	(Psychiatr* NEAR/3 (Care OR Service OR Center OR Hospital OR Facilit* OR Institution OR System OR Plan OR Program* OR Policy OR Organization OR Organisation OR Needs OR Insurance OR Disparities OR Ward OR Unit OR Clinic OR Department OR Model OR Strateg*)):ti,ab,kw	3,879
S14	S4 OR S5 OR S6 OR S7 OR S8 OR S9 OR S10 OR S11 OR S12 OR S13	66,157
S15	S3 AND S14	1,044
	S15 AND filters activated: Publication year: 1990 – today	1,044

MEDLINE 230814

S1	MH "Iran"	37,444

S2	TI (iran OR iranian OR kurdistan OR kurdish OR balochistan OR baluchistan OR baluchestan OR lorestan OR luristan OR lurestan OR loristan) OR AB (iran OR iranian OR kurdistan OR kurdish OR balochistan OR baluchistan OR baluchestan OR lorestan OR luristan OR lurestan OR loristan)	66,963
S3	S1 OR S2	73,330
S4	MH "Mental Health Services+"	105,644
S5	MH "Psychiatry+"	110,634
S6	MH "Psychotherapy"	57,871
S7	MH "Psychiatric Nursing"	18,236
S8	MH "Psychiatric Department, Hospital"	7,005
S9	MH "Hospitals, Psychiatric"	26,068
S10	MH "Community Mental Health Centers+"	3,320
S11	TI ((mental OR socio-mental OR behavioral) N3 ("health care" OR "health services" OR "health center" OR "health hospital" OR "health facilit*" OR "health institution" OR "health system" OR "health plan" OR "health program*" OR "health policy" OR "health organization" OR "health organization" OR "health needs" OR "health insurance" OR "health disparities" OR "health ward" OR "health unit" OR "health clinic" OR "health department" OR "health model" OR "health strateg*")) OR AB ((mental OR socio-mental OR behavioral) N3 ("health care" OR "health services" OR "health center" OR "health hospital" OR "health facilit*" OR "health institution" OR "health system" OR "health plan" OR "health program*" OR "health policy" OR "health organization" OR "health organization" OR "health needs" OR "health insurance" OR "health disparities" OR "health ward" OR "health unit" OR "health clinic" OR "health department" OR "health model" OR "health strateg*"))	48,912
S12	TI (counseling OR counselling OR "behavioral therap*" OR "behavioural therap*" OR "cognitive behavioral therap*" OR "cognitive behavioural therap*" OR psychotherap* OR "psychodynamic therap*") OR AB (counseling OR counselling OR "behavioral therap*" OR "behavioural therap*" OR "cognitive behavioral therap*" OR "cognitive behavioural therap*" OR psychotherap* OR "psychodynamic therap*")	177,497
S13	TI (Psychiatr* N3 (Care OR Service OR Center OR Hospital OR Facilit* OR Institution OR System OR Plan OR Program* OR Policy OR Organization OR Organisation OR Needs OR Insurance OR Disparities OR Ward OR Unit OR Clinic OR Department OR Model OR Strateg*)) OR AB (Psychiatr* N3 (Care OR Service OR Center OR Hospital OR Facilit* OR Institution OR System OR Plan OR Program* OR Policy OR Organization OR Organisation OR Needs OR Insurance OR Disparities OR Ward OR Unit OR Clinic OR Department OR Model OR Strateg*))	60,363
S14	S4 OR S5 OR S6 OR S7 OR S8 OR S9 OR S10 OR S11 OR S12 OR S13	466,973
S15	S3 AND S14	1,745
	S15 AND filters activated: Publication year: 1990 – today, Language: English	1,713

Scopus 230815

S 1	TITLE-ABS-KEY (iran OR iranian OR kurdistan OR kurdish OR balochistan OR baluchistan OR baluchestan OR lorestan OR luristan OR lurestan OR loristan)	21 2 49 9
S 2	TITLE-ABS-KEY ((mental OR socio-mental OR behavioral) W/3 ("health care" OR "health services" OR "health center" OR "health hospital" OR "health facilit*" OR "health institution" OR "health system" OR "health plan" OR "health program*" OR "health policy" OR "health organization" OR "health organization" OR "health needs" OR "health insurance" OR "health disparities" OR "health ward" OR "health unit" OR "health clinic" OR "health department" OR "health model" OR "health strateg*"))	15 2 52 7
S 3	TITLE-ABS-KEY (counseling OR counselling OR "behavioral therap*" OR "cognitive behavioral therap*" OR psychotherap* OR "psychodynamic therap*")	46 2 74 7
S 4	TITLE-ABS-KEY psychiatr* W/3 (care OR service OR center OR hospital OR facilit* OR institution OR system O R plan OR program* OR policy OR organization OR organisation OR needs OR insurance OR disparities OR ward OR unit OR clinic OR d epartment OR model OR strateg*)	10 5 23 7

S 5	S2 OR S3 OR S4	66 6 67 5
S 6	S1 AND S5	3 21 1
S 7	S6 and filters activated: Year 1990-2023, English	2 95 8

Web of Science 230815

S1	iran OR iranian OR kurdistan OR kurdish OR balochistan OR baluchistan OR baluchestan OR lorestan OR luristan OR lurestan OR loristan (Topic)	144 827
S2	Mental NEAR/5 (care or service or system or ward or hospital or unit or clinic or department or program*)	62 831
S3	counseling OR counselling OR "behavioral therap*" OR "cognitive behavioral therap*" OR psychotherap* OR "psychodynamic therap*" (Topic)	231 427
S4	psychiatr* NEAR/3 (care OR service OR center OR hospital OR facilit* OR institution OR system OR plan OR program* OR policy OR organization OR organisation OR needs OR insurance OR disparities OR ward OR unit OR clinic OR department OR model OR strateg*) (Topic)	51 816
S5	S2 OR S3 OR S4	328 650
S6	S1 AND S5	1 841
S7	S6 and filters activated: Publication years 1990-2023, English	1 827
