# Supplementary material for: Mental Health and Mental Health Care in Iran: Addressing Social Inequalities
Source: Healthcare (Basel). 2025 Dec 1;13(23):3131. doi: 10.3390/healthcare13233131 (PMC12692221; doi:10.3390/healthcare13233131)
Supplement: Supplementary file 1 [file healthcare-13-03131-s001.zip › healthcare-3907829-supplementary/Additional file S2.pdf]

Peer-reviewed journal articles used in the Results section

Authors	Year	Title	Design	Aim	Findings	Evidence Levels	Quality Ratings
Hajebi A, Damari B, Vosoogh Moghaddam A, Nasehi A, Nikfarjam A, Bolhari J.	2013	What to do to promote mental health of the society	Analytical review and Focus Group Discussions	To prepare national policy and interventions for promoting mental health.	The prevalence of mental disorders in the last decade has increased. Coverage of mental health programs in two last decades in the best could be equal to rural population. Urban areas have been deprived of these services.	III	B
Hosseini Jebeli et al.	2021	Scaling-up a new socio-mental health service model in Iran to reduce burden of neuropsychiatric disorders: an economic evaluation study	An economic evaluation following One Health Tool methodology	To estimate the impact of scaling-up the new socio-mental health model at the national level as well as its associated costs.	The health impacts are calculated in terms of healthy life years gained for 2020-30, after adjusting the prevalence and incidence rates for each disorder.	II	B
Farshad Farzadfar, Mohsen m, et.al.	2019	Health system performance in Iran: a systematic analysis for the Global Burden of Disease Study 2019	Systematic analysis	To provide a comprehensive national and subnational breakdown using the Global Burden of Diseases, Injuries, and Risk Factors Study (GBD)	Iran had a life expectancy of 79·6 years. The number of DALYs remained stable and reached 19·8 million in 2019, of which 78·1% were caused by non-communicable diseases.	III	B
Behzad Damari, Vandad Sharifi, Mohammad Hossein Asgardoon, Ahmad Hajebi	2021	Iran’s Comprehensive Mental and Social Health Services (SERAJ Program): A Pilot Protocol.	Action research - review of the literature, interviews and group discussions	To develop and outline the protocol for the pilot implementation of social and mental health services, entitled "SERAJ".	The pilot implementation should be divided into 4 phases: preparing documentation and work team, preparing the prerequisites for providing pilot services, providing pilot services, and implementation and evaluation.	III	B
Rahimi-Movaghar A, Amin-Esmaeili M, Sharifi V, Hajebi A, Radgoodarzi R, Hefazi M, et al.	2014	Iranian mental health survey: design and field proced	A cross-sectional national household survey with face-to-face interviews as	To assess the twelve-month prevalence and severity of psychiatric disorders in the Iranian	An overview of the study design, fieldwork organization and procedures, weightings and analysis.	III	B

Authors	Year	Title	Design	Aim	Findings	Evidence Levels	Quality Ratings
			the main data collection method	adult population and to determine the pattern of health care utilization and cost of services.			
Behzad Damari, Siamak Alikhani, Sahand Riazi-Isfahani, Ahmad Hajebi.	2017	Transition of Mental Health to a More Responsible Service in Iran	Review of literature - using inputs from the stakeholders and experts.	To propose a model for provision of an effective universal coverage for mental health services	The new model included 2 basic and specialized service strata: a PHC-based infrastructure and essential requirements needed to establish the service.	III	B
Sharifi V, Abolhasani F, Farhoudian A, Amin-Esmaeili M.	2014	Community mental health centers in Iran: planning evidence-based services	through an evidence-based approach - systematic review approach,	To present the process of designing structure and planning the services for CMHCs	Depression, schizophrenia and other psychotic disorders, bipolar disorders and anxiety disorders respectively comprise the top four psychiatric disorders, according to burden of diseases, in Iran. Urban areas have richer resources than rural areas in the country, though there are no out-of-hospital and out-of-office mental health services and the network system and the primary care for presenting mental health services are ineffective due to the current restrictions.	III	B
Sharifi V. et.al.	2016	Child and Adolescent Mental Health Care in Iran: Current Status and Future Directions	Literature review	To provide an overview of the extent of unmet need and mental health services in Iran.	Child and adolescent mental and behavioral health problems are highly prevalent in Iran. Different studies have estimated that 16.7% to 36.4% of children and adolescents suffer from one or more mental health problems. However, there is a serious scarcity of resources to meet this need.	III	B

Authors	Year	Title	Design	Aim	Findings	Evidence Levels	Quality Ratings
					Available specialized child and adolescent services are mostly confined to small inpatient units and university outpatient facilities in larger cities, and there is a scarce evidence for the effectiveness of the available services.		
Forouzan Setareh, Padyab Mojgan, Rafiey Hassan, Ghazinour Mehdi, Dejman Masoumeh, San Sebastian Miguel	2016	Measuring the Mental Health-Care System Responsiveness: Results of an Outpatient Survey in Tehran	Survey - questionnaire	To measure responsiveness and its related domains in the mental health-care system of Tehran	On average, 47% of participants reported experiencing poor responsiveness. Among responsiveness domains, confidentiality and dignity were the best performing factors while autonomy, access to care, and quality of basic amenities were the worst performing. Respondents who reported their social status as low were more likely to experience poor responsiveness overall.	II	B
Roozbeh, N., Sanati, A., Abdi, F.	2018	Afghan refugees and immigrants health status in Iran: a systematic review	Systematic review	To provide reliable information on Afghan refugees' health status in Iran	The results demonstrate high prevalence of both communicable and non-communicable disorders as well as psychological problems in this population.	III	B
Riahi, A., Hariri, N., Nooshinfard, F.	2015	Study of Health Information Needs and Barriers to Access among Afghan and Iraqi Immigrants in Iran	Survey-questionnaires	To study needs and barriers for accessing health information among Afghan and Iraqi Immigrants in Iran.	General Health Information mentioned as the most important health information needs for immigrants. Family, Friends and other immigrants were also most important source of access to information for them. Health knowledge	III	B

Authors	Year	Title	Design	Aim	Findings	Evidence Levels	Quality Ratings
					and information of foreign immigrants in Iran were in low status.		
Moradpour, F., Hajebi, A., Salehi, M., Solaymani-Dodaran, M., Rahimi-Movaghar, A., Sharifi, V., Amin-Esmaeili, M., Motevalian, S. A.	2019	Province-Level Prevalence of Psychiatric Disorders: Application of Small-Area Methodology to the Iranian Mental Health Survey (IranMHS)	Mental Health Survey	To provide province-level estimates of psychiatric disorders.	A wide variation in the prevalence of psychiatric disorders was found among 31 provinces of Iran. The direct estimates ranged from 3.6% to 62.6%, while the HB estimates ranged from 12.6% to 36.5%. The provincial prevalence among men ranged from 11.9% to 34.5%, while it ranged from 18.4% to 38.8% among women.	II	B
E. Barfar, A. Pourreza, V. Sharifi, S. M. H. Sobhanian and A. A. Sari	2019	Catastrophic Health Expenditure in Households with Severe Mental Disorders Patients: Evidence After Iran's Health Transformation Plan	A cross-sectional study - World Health Survey questionnaire through interviews.	To measure the catastrophic health expenditure (CHE) for households with severe mental disorders (SMDs) patients.	There was a significant relationship between households facing CHE and variables including the age of the household head, education status of the household head, utilization of dental, rehabilitation and medication, and the household expenditures quintile. It is suggested that the Health Transformation Plan financially covers outpatient healthcare services more sufficiently, especially for mental disorders.	II	B
M. Tabatabaee, Z. Mirsepassi, V. Sharifi and Y. Mottaghipour	2021	Challenges of implementing psychiatric rehabilitation services: a low- and middle-income country case example	Mixed-methods - evaluation by adopting a multi-method approach	To explore the barriers and challenges of implementing psychiatric rehabilitation at a psychiatric hospital.	A low participation rate, administrative issues, low fidelity to protocols and incomplete documentation were the main findings.	II	B

Authors	Year	Title	Design	Aim	Findings	Evidence Levels	Quality Ratings
Irajpour, M. Alavi, S. Abdoli and M. B. Saberizafarghandi	2012	Challenges of interprofessional collaboration in Iranian mental health services: A qualitative investigation	Qualitative study Interviews	To explore interprofessional collaboration to improve mental health services.	Some important challenges were identified as protecting professional territory, medical oriented approach and teamwork deficits. It could shed insight into underlying causes of collaboration gaps among nurses and other health professionals.	III	B
H. Zarafshan, L. S. Wissow, Z. Shahrivar, R. Mojtabai, M. Khademi, M. JafariNia, et al.	2021	Children and adolescents' mental health in Iran's primary care: Perspectives of general practitioners, school staff and help seekers	Qualitative - Interviews	To obtain information relevant to the design of a training program for general practitioners (GPs) to extend this collaboration to include services for children and adolescents.	GPs reported need for additional training in diagnosis and management, especially in skills for interviewing and communicating with children. Adolescents expressed a preference not to speak about private issues in the presence of their parents, and expressed concern that the GPs did not respect their preference. They also desired a more active role during visits.	III	B
H. Khazaie, F. Najafi, B. Hamzeh, A. Chehri, A. Rahimi-Movaghar, M. Amin-Esmaeili, et al.	2018	Cluster analysis of psychiatric profile, its correlates, and using mental health services among the young people aged 15–34: Findings from the first phase of Iranian youth cohort in Ravansar	Persian Youth Cohort. Interviews, using questionnaire	(1) Cluster analysis of psychiatric disorders and partitioning the youth; (2) determining socio-demographic correlates and parental histories for each one of the clusters; and (3) comparing clusters based on the extent and type of using psychotherapeutic services.	There is a direct relationship between widow/divorced marital status and psychiatric maternal history with the clinical cluster. Clinical and non-clinical clusters with medium to severe disability used services for mental health more often that the healthy cluster with mild functional disability.	II	B
Y. Azizpour, K. Asadollahi, K. Sayehmiri,	2016	Epidemiological survey of intentional poisoning suicide during 1993-	Retrospective study - data from intentional	To epidemiologically assess committing suicide in Ilam province, Iran,	Totally, 6794 cases of suicide associated with poisoning were evaluated. The incidence rate	II	B

Authors	Year	Title	Design	Aim	Findings	Evidence Levels	Quality Ratings
S. Kaikhavani and G. Abangah		2013 in Ilam Province, Iran	poisoning suicide in Ilam Province	based on intentional poisoning.	of suicide attempts was (94.51 in female and 74.98 in male) and the incidence rate of completed suicide was (1.94 in female and 2.40 in male). Also, the highest rates of attempted and completed suicide (annual incidence rate of 172.42 and 4.14, respectively) were attributed to the age group of 15-24 year.		
M. Amin-Esmaeili, A. Rahimi-Movaghar, V. Sharifi, A. Hajebi, R. Radgoodarzi, R. Mojtabai, et al.	2016	Epidemiology of illicit drug use disorders in Iran: prevalence, correlates, comorbidity and service utilization results from the Iranian Mental Health Survey	Mental Health Survey (IranMHS) - Face-to-face interviews	To provide prevalence estimates and information on correlates of illicit drug use disorder and opioid dependence, as well as service use for these disorders in Iran	Opioid use disorders, and opium in particular, were the most common use disorder. The odds of drug use disorders were greater in men than in women, in previously married participants than in currently or never married participants, and in participants with lower socio-economic status than in those with higher socio-economic status.	II	B
M. Bahrami, A. Jalali, A. Ayati, A. Shafiee, F. Alaedini, S. Saadat, et al.	2023	Epidemiology of mental health disorders in the citizens of Tehran: a report from Tehran Cohort Study	Survey – Interviews and Questionnaire	To evaluate the epidemiology of mental health disorders in the citizens of Tehran using the Tehran Cohort Study (TeCS) data	Almost 37.1% of Tehran residents suffered mental health problems (45.0% of women and 28.0% of men). The greatest incidence of MHDs was seen in the 25-34 and over 75 age groups. The most common mental health disorders were depression (43%) and anxiety (40%), followed by somatization (30%) and social dysfunction (8.1%).	III	B

Authors	Year	Title	Design	Aim	Findings	Evidence Levels	Quality Ratings
P. Safavi, M. R. Mohammadi, A. Khaleghi, S. A. Mostafavi, S. Taheri, K. Shahbazi, et al.	2019	Epidemiology of Psychiatric Disorders in Children and Adolescents in Chaharmahal and Bakhtiari Province, Iran	Community-based cross-sectional study - Interviews	To investigate the epidemiology of psychiatric disorders in children and adolescents in Chaharmahal and Bakhtiari Province of Iran	A total of 16.1% of participants were diagnosed to have psychiatric disorders. Total psychiatric disorders were significantly more prevalent in boys than in girls.	II	B
F. Najafi, Y. Pasdar, B. Karami Matin, S. Rezaei, A. K. Karyani, S. Soltani, et al.	2020	Decomposing socioeconomic inequality in poor mental health among Iranian adult population: Results from the Persian cohort study	Baseline data of Persian cohort study	To assess socioeconomic inequality in poor mental health among Iranian adults.	The estimated E for poor mental health indicating slightly higher concentration of mental health problem among socioeconomically disadvantaged adults in Iran. Socioeconomic inequality in poor mental health was mainly explained by gender (19.93%) and age (12.70%). Region, SES and physical activity were other important factors that contributed to the concentration of poor mental health among adults with low socioeconomic status.	II	B
E. Homaie Rad, L. Amirbeik, M. Hajizadeh, S. Yousefzadeh-Chabok, Z. Mohtasham-Amiri, S. Rezaei, et al	2020	Determinants of utilization and out-of-pocket payments for psychiatric healthcare in Iran	Cross-sectional study - Household Income and Expenditure Survey (HIES)	To assess the determinants of the utilization and out-of-pocket payments (OOP) for psychiatric healthcare in Iran.	The average of utilization and OOP for psychiatric services was found to be 14.67 times per 1,000 households and $7.783 per month for service users, respectively. There were significant positive relationships between income and utilization. Significant negative associations were found between the number of illiterate people in the household and OOP. Utilization and OOP for psychiatric services were	II	B

Authors	Year	Title	Design	Aim	Findings	Evidence Levels	Quality Ratings
					statistically significantly higher among households with higher wealth status.		
K. Keshavarz, A. Hedayati, M. Rezaei, Z. Goudarzi, E. Moghimi, M. Rezaee, et al.	2022	Economic burden of major depressive disorder: a case study in Southern Iran	Cross-sectional - census method - calculate the costs using patients' medical records and insurance bills as well as their self-reports or those of their companions	To determine the economic burden of Major Depressive Disorder (MDD) on the patients referred to the reference psychiatric single-specialty hospitals in southern Iran in 2020	The annual cost of MDD was $ 2717.41 Purchasing Power Parity (PPP) per patient in 2020. Direct medical costs accounted for the largest share of the costs (73.68%), of which hoteling and regular beds expenses were the highest (57.70% of the total direct medical costs). The shares of direct non-medical and indirect costs were 7.52 and 18.80%, respectively, and the economic burden of the disease in the country was estimated at $7,120,456,596 PPP.	II	B
K. Zarea, A. Nikbakht-Nasrabadi, A. Abbaszadeh and A. Mohammadpour	2012	Facing the challenges and building solutions in clinical psychiatric nursing in Iran: a qualitative study	Qualitative approach-Semistrucutred interviews	To understand the perspectives of psychiatric nurses regarding the issues they face while providing care and examine the possible solutions for improvement of inpatient care in clinical settings.	The first, Challenges in Providing Care within Psychiatric Wards, had the following subthemes: Politics and Rules of Organization, Safety and Security Issues, Uncertainty about the Role, Lack of Trained Staff, and Sociocultural Issues. The second theme, Solutions for Improving Psychiatric Care, had the subthemes of Empowerment across four domains: Psychiatric Nurses, Mentally Ill Patients and their Families, The Psychiatric Mental Health System, and the Cultural Context.	III	B

Authors	Year	Title	Design	Aim	Findings	Evidence Levels	Quality Ratings
H. Kakaei, F. Maleki, A. Biderafsh, R. Valizadeh, M. A. Mansournia, I. Pakzad, et al.	2023	High prevalence of mental disorders: a population-based cross-sectional study in the city of Ilam, Iran	population-based cross-sectional study - Questionnaire	To determine the age- and sex-standardized prevalence and risk factors of depression, anxiety, and stress symptoms in the city of Ilam	The age- and sex-standardized prevalence of severe depression, anxiety, and stress symptoms was 19.90%. There was a positive association among depression and anxiety symptoms with female sex and Kurdish ethnicity, low educational level, job losing history, mental disorders history, hopelessness for the future, and history of other diseases.	II	B
K. Mohamadi, K. Ahmadi, A. Fathi Ashtiani, P. Azad Fallah, A. Ebadi and E. Yahaghi	2014	Indicators of mental health in various Iranian populations	Q-methodology which combines both quantitative and qualitative methods	To select a set of mental health indicators that can be used to monitor the status of mental health in Iran	The most important indicators: annual prevalence of mental disorders, suicide rates, number of mental health professionals, mental health expenditures and suicide related deaths.	II	B
Hajebi A, Sharifi V, Abbasinejad M, Asadi A, Jafari N, Ziadlou T, Khadem S, Asgardoon MH, Damari B.	2021	Integrating Mental Health Services into the Primary Health Care System: The Need for Reform in Iran	Literature review, deep interview with stakeholders, and focused group discussions	To review aspects of the current situation and structure of the integrated mental health care services for planning a reform.	The main gaps of the public mental health care (PHC) services in Iran were identified, which included reduction of risk factors for mental disorders, training the general population, early recognition and treatment of patients with mental disorders, educating patients and their families, and rehabilitation services.	III	B
M. B. Eftekhari, A. S. Forouzan, A. Mirabzadeh, H. Sajadi, M. Dejman, H. Rafiee, et al.	2014	Mental health priorities in Iranian women: overview of social determinants of mental health	Community based participatory research - Qualitative	To describe mental health priorities in Iranian women and overview social determinants of mental health	According to the qualitative phase, the first mental health priority in socioeconomic level was lack of insurance for unattended families, it was unemployment in differential	III	B

Authors	Year	Title	Design	Aim	Findings	Evidence Levels	Quality Ratings
					exposure level and it was lack of knowledge and skills related to dealing with stress in the differential vulnerability level; in differential outcome and consequence levels, the priorities were lack of free counseling centers in the study area and lack of facilities for mental health rehabilitation.		
M. Shirzadi, H. Khazaie and S. Farhang	2017	Mental health survey of adult population in Kermanshah County, 2015: Preliminary report	General health questionnaire (GHQ)	To perform a population-based study about mental health in West of Iran.	This population was 656683 from whom 83.6% lived in urban area and 16.4% lived in rural area. A positive result was achieved from 39.1% (out of 2102). It was not related to gender, age group, job or marital status but illiterate participants, and those with lower economic level had higher scores.	II	B
H. Zarafshan, M. R. Mohammadi and M. Salmanian	2015	Prevalence of Anxiety Disorders among Children and Adolescents in Iran: A Systematic Review	Literature review	To conduct a review to investigate the prevalence of anxiety disorders among Iranian children and adolescents	Results show different types of anxiety disorders. All anxiety disorders were mostly investigated with the prevalence rates ranging from 6.8% in Saravan to 85% in Bandar Abbas. OCD was the second common study with prevalence rates ranging from 1% in Tabriz to 11.9% in Gorgan.	III	B
D. Sarokhani, M. Parvareh, A. Hasanpour Dehkordi, K. Sayehmiri and A. Moghimbeigi	2018	Prevalence Depression among Iranian Elderly: Systematic Review and Meta-Analysis	Literature review - Questionnaire	To estimate the prevalence of depression among the Iranian elderly through meta-analysis method	Prevalence of depression among Iranian elderly was estimated to be 43%. The findings showed that the prevalence of depression	II	B

Authors	Year	Title	Design	Aim	Findings	Evidence Levels	Quality Ratings
					among Iranians were 49% in women, 48% in men, 37% in unmarried, and 45%in the married. In addition, the prevalence of very severe, severe, moderate, and mild depression levels was estimated to be 5%, 19%, 33%, and 38% of the participants, respectively.		
B. Moeini, S. Bashirian, A. R. Soltanian, A. Ghaleiha and M. Taheri	2019	Prevalence of depression and its associated sociodemographic factors among Iranian female adolescents in secondary schools	Cross-sectional study-Questionnaire	To screen the prevalence of depression among secondary school female students in the city of Hamadan, in western Iran.	The mean age of students was 16.2 years. The prevalence of severe depression in female students was equal to 52.6%. A statistically significant relationship was also observed to exist between prevalence of depression and type of school, family income, living in the suburbs, and field of study at school.	III	B
M. Seyf Hashemi, E. Yarian, P. Bahadoran, J. Jandaghi and M. Mirmohammad Khani	2015	Prevalence of Mental Health Problems in Children and Its Associated Socio-Familial Factors in Urban Population of Semnan, Iran (2012)	Random cluster sampling-questionnaire	To screen Mental Health Problems (MHP) and its impact in an Iranian urban population aged 6 - 12 years old, and explored its associated socio-familial factors	The frequency of problems ranged between 16.1% (peer problems) and 8.4% (emotional symptoms), and in all subscales boys were affected more than girls. The impact score was abnormal in 68.4% of all children, and was greater in girls than in boys.	II	B
M. R. Mohammadi, S. S. Alavi, N. Ahmadi, A. Khaleghi, K. Kamali, A. Ahmadi, et al.	2019	The prevalence, comorbidity and socio-demographic factors of depressive disorder among Iranian children and adolescents: To	cross-sectional study-Questionnaire	To identify the main risk factors that can affect the incidence of depression in Iranian children and adolescents.	Age (15–18), being female, and the father's unemployment were associated with an increased odds ratio for depressive disorders. Depressive symptoms are common in children and	III	B

Authors	Year	Title	Design	Aim	Findings	Evidence Levels	Quality Ratings
		identify the main predictors of depression			adolescents and are correlated with age and gender.		
M. F. Khesht-Masjedi, S. Shokrgozar, E. Abdollahi, B. Habibi, T. Asghari, R. S. Ofoghi, et al.	2019	The relationship between gender, age, anxiety, depression, and academic achievement among teenagers	The Beck Anxiety Inventory and The Beck Depression Inventory	To identify the impact of anxiety and depression on academic achievement in students living in North of Iran	Girls with 21.8% were more anxious than boys with 11.6%, while boys with 29.5% are more depressed than girls with 17.8%. There was no significant difference in the mean of anxiety and age between teenagers, but a significant depression level of respondents who are 18 and 19 years old was significantly different from other ages.	II	B
Z. Mahmoodi, M. Dolatian, A. Mirabzadeh, H. A. Majd, F. Moafi and M. Ghorbani	2017	The relationship between household socioeconomic status and mental health in women during pregnancy: A path analysis	Demographic and obstetrics questionnaire	To investigate the relationship between household socioeconomic status and mental health in women during pregnancy in 2014 - 2015	Social support had the greatest negative effect, however household size had the greatest positive effect on maternal mental health. Paternal education and income had the greatest negative effects and maternal education the greatest positive effect on maternal mental health.	II	B
M. Taban, A. Hajebi, M. Gholami and M. Naserbakht	2021	Research paper evaluating the performance of community mental health centers in Iran: Strengths and challenges	Qualitative study-interviews	To evaluate the performance of Community-based Mental Health Centers (CMHCs) in Iran and identifying the strengths and challenges in the provision of mental health services.	The findings were presented in terms of the needs for program implementation, achieving the expected goals and outcomes, and the findings of program evaluation. Challenges in the implementation of this program included the barriers to attracting more general physicians and the impact of culture and attitudes in each city on the effectiveness of the program.	III	B

Authors	Year	Title	Design	Aim	Findings	Evidence Levels	Quality Ratings
A. A. Noorbala, S. A. Bagheri ayati, S. Faghihzadeh, K. Kamali, E. Faghihzadeh, A. Hajebi, et al.	2017	A survey on mental health status of adult population aged 15 and above in the province of Chaharmahal and Bakhtiari, Iran	Cross-sectional field survey	To determine the mental health status of population aged 15 and over in the province of Chaharmahal and Bakhtiari in 2015	Urban areas were more at risk of mental disorders compared with rural residents. Anxiety and somatization symptoms were more frequent than depression and social dysfunction among respondents. The prevalence of mental disorders increased with age. Mental disorders were more common among females, people aged 65 years and above, the divorced and widowed, illiterate and unemployed adults.	II	B
Pirdehghan A, Poor Rezaee M, Mirzababaee B	2017	Epidemiology of Substance Abuse Among Iranian Adolescents (Yazd: 2014)	cross-sectional study-Questionnaire	To estimate the prevalence of substance abuse and evaluating some of its associated factors in Iranian adolescents	Hookah was the most common substance used by students, with 41.1% trying it once and 31.1% using it regularly. Statistical models identified key predictors for use: male gender, studying mathematical physics, and prior hookah/alcohol use were significant for regular smoking; male gender and prior cigarette/hookah use predicted alcohol use; and male gender, studying mathematics, poor educational performance, high father education, and prior tranquilizer use were predictors for illicit drug use.	II	B
V. Farnia, J. Shakeri, T. A. Juibari, F. Tatari, M. Khoshbakht and A. Aghaei.	2016	Demographic and clinical features of patients with substance-induced mental disorders	Cross-sectional study-questionnaire and clinical exams	To evaluate demographic and clinical features of patients with substance induced mental disorders	The most of the patients were unemployed males with low education living in urban areas. Amphetamines (80.5%),	II	B

Authors	Year	Title	Design	Aim	Findings	Evidence Levels	Quality Ratings
		admitted to the psychiatric hospital in Kermanshah, Iran.		admitted to Farabi psychiatric hospital in Kermanshah, Iran	opioids (15.8%), cannabis (3.1%), and benzodiazepines in (0.6%) patients were the main drugs leading admission. Amphetamine induced psychotic disorder with hallucination (40.4%) were the most common diagnosis among the study subjects.		
Mianji F, Kirmayer LJ.	2021	"Women as Troublemakers": The Hard-Sociopolitical Context of Soft Bipolar Disorder in Iran	Qualitative study, in-depth semi-structured interviews	To discuss the gendered meanings of the diagnosis of Bipolar Spectrum Disorder (BSD) in Iran.	Findings suggest that the high rate of diagnosis of bipolar spectrum disorder among women in Iran is influenced by gender, sociocultural, political, and economic factors, as well as the diagnostic practices of biomedical psychiatry.	III	B
Shahabi S., Maharlouei N., Joulaei H., Bagheri Lankarani K.	2021	Improving the Women's Mental Health Status in Iran: A Policy Brief	Content analysis of conference	Providing suitable policy solutions in order to improve women's mental health in Iran	Women's mental health status in Iran is not desirable. It implies the importance of preventive interventions and improving mental health services. Other strategies could be following recommendations provided to promote women's mental health in Iran including improving primary health care by mental health services, assigning a specified authority to enhance the intra-sectoral governance and intersectoral leadership.	V	C
Neamatshahi, M. Ebrahimipour H., keyvanlo Z., Khajedaluee M., Keykhosravi A.	2020	Assessment of Health Budget during the First to Fifth Socioeconomic Developmental Programs in Iran	Literature review of books “Law of the country budget" in four socioeconomic	To evaluate the health budget trend and allocation of the total budget to it during the first	Despite the growth in health budget from the first to the fourth program, the allocated budget to health was constantly decreasing compared to the	IV	C

Authors	Year	Title	Design	Aim	Findings	Evidence Levels	Quality Ratings
			developmental programs	to fourth socioeconomic developmental programs	other sections such as industry and so on. Therefore, policy makers and planners should pay more attention to allocation of resources to health.		
Khavari K, Sajadi H, Habibpour Gatabi K, Talebi M.	2013	Gender Inequality Perception and Mental Health	Quantitative- Correlation method	To investigate the relationship between the dimensions of feeling gender inequalities and the mental health of working women	There was a positive and significant relationship between the feeling of gender inequality and mental health; So, the less the feeling of inequality, the better the mental health of people.	III	B
Mehrdad Mohammadian, Mahboobeh Karami, Jafar Bolhari, Eisa Karimi	2013	Screening of mental disorders in Afghan immigrants living in Tehran	A quantitative study – Descriptive - Cross-sectional study	Screening of mental disorders of Afghan immigrants living in Tehran in 2013	The results showed that the prevalence of mental disorders in Afghan immigrants is 55.6% (19.9% men and 35.7% women). In this study, a significant relationship was found between mental disorders and demographic characteristics, except for the number of people in the household. There was a significant relationship between mental disorders and the way of residence.	II	B
Jafar Bolhari, Kourosh Kabir, Ahmad Hajebi, Seyed Abas Bagheri Yazdi, Hasan Rafiei, Masoud Ahmadzad Asl, Nilofar Mahdavi Hazave, Mohamadreza Rahbar, Seyed Abbas Motevalian, Hosein Kazemaini	2016	Revision of the Integration of Mental Health into Primary Health care and the Family Physician Program	A combination of review literature and qualitative methods Group discussion, expert panels and Delphi technique	Introducing the goals, strategies and necessary measures set in the revision of the mental health integration program in primary health care and the family Physician program.	The program's intended values in several domains and with specifying the general goal, were formed, and to achieve these goals, strategies in various areas including service delivery, training, information system, evaluation, advocacy, provision of essential medicines, quality	III	C

Authors	Year	Title	Design	Aim	Findings	Evidence Levels	Quality Ratings
					improvement, and financial and administrative affairs were designed. For each strategy, objectives and measures were identified.		
Hosseini Shokouh, S. M., Arab, M., Emamgholipour, S., Meskarpour, A. M.	2021	Socio-Economic Inequality and Mental Health in Tehran	Cross-sectional descriptive - analytical study	To investigate the relationship between socio-economic factors and mental health of households living in Tehran.	Among the socioeconomic variables, four variables including years of education, health behaviors (regular physical activity, healthy eating habits), not-smoking, and social capital had a positive and significant relationship with all mental health measures (social role, emotional role, mental health and vitality).	II	B
Mansoureh Khalili, Shahram Vaziri, Farah Lotfi Kashani	2022	Mental Health Services for Transgender People: A Systematic Review Study	Systematic Review	To examine the mental health services and needs of transgender people and their attitudes and experiences	Some of main challenges of transgender include access to health services tailored to the needs of transgender people and the need to develop mental health services for these people. Many transgender people refuse treatment because of prejudices and irrelevant questions of the medical service providers, unnecessary tests, restrictive attitudes of the treatment staff, restrictive suggestions and treatment methods.	III	B
Esmaeil Khedmati Morasae, Mohsen Asadi Lari, Ameneh Setareh Forouzan, Reza Majdzadeh, Mehdi Mirheidari, Hamid Nabavi	2012	Avoidable socioeconomic inequality in mental health distribution in Tehran: Concentration	The Concentration index (C) approach was applied to examine socioeconomic	To measure socioeconomic inequality in distribution of mental disorders in Tehran	Concentration index of mental health showed that, there is inequality unfavorable to poorer people in Tehran respecting mental disorders. Since much of the observed	II	B

Authors	Year	Title	Design	Aim	Findings	Evidence Levels	Quality Ratings
		Index standardization approach	inequality in mental health in Tehran		inequality in mental health is probably due to social factors and not to demographic ones, therefore the possibility of inequality correction and its avoidance increase.		
Mohammad Tavakkol, Ebrahim Ekhlasi, Seyyed Pouya Rasoulinejad	2020	Systematic review of studies on the determinants of mental health in Iranian society Exploration case: Internal scientific research articles 1399-1385	Systematic review	To identify and classify the variables related to mental health in Iranian society.	Results show that mental health is affected by 36 variables in four types of social, cultural, contextual and psychological, the product of social and cultural conditions. Social variables have the highest frequency in 36.6% of cases and psychological variables in 23.4% of cases have the least importance among the authors of the reviewed studies.	III	B
Smith, A. K.	2020	The Integration of Mental Health Care in Rural Iran	Review - Essay	To describe the integration of mental health care in rural areas	The integration of mental health care led to the rapid improvement of health outcomes. The integration also created the unintended consequence of privileging pharmaceutical treatments and overlooking mental illnesses affected by somatization.	V	C
Dorosti, A., Karamouz, M., Asl Rahimi, V., Azimzadeh, S., Gharaee, H., Azami-Aghdash, S., Farahbakhsh, M.	2023	Mental Health Services Integration in Primary Health Care in Iran: A Policy Analysis	Qualitative research - using a case study approach	To analyze the policy of integrating N-MHSs in PHC, focusing on the analysis of the current situation, pathology, and the existing challenge.	Twenty weaknesses were extracted in eight areas, including negative views of mental health, weaknesses in human resource training, compensation for the service of psychologists, unfavorable working conditions of the workforce, inappropriate	III	B

Authors	Year	Title	Design	Aim	Findings	Evidence Levels	Quality Ratings
					service delivery facilities, lack of meaningful communication between different levels of service delivery, poor inter-sectorial communication, and the challenging nature of mental health care.		
Damari, B., Mafimoradi, S.	2019	Intersectoral expectations for promoting mental health: a qualitative case study of Islamic Republic of Iran	Reviewing the literature, interviewing with experts, and holding focus group discussions.	To determine the role of national organizations in promoting mental health in Iran.	The roles of 31 national organizations in five groups were determined. Then, the intersectoral organizational expectations in two key areas were extracted. The key roles determined were mostly related to the Ministry of Education, Islamic Republic of Iran Broadcasting, Ministry of Labor and Social Welfare, and the Ministry of Sports and Youth.	III	B
Veisani, Y., Mohamadian, F., Delpisheh, A.	2017	Prevalence and comorbidity of common mental disorders and associations with suicidal ideation in the adult population	Cross-sectional study - cluster random sampling	To examine the relationship between the presence of comorbid mental disorders and suicidal ideation in the adult population.	Major depressive disorder and obsessive-compulsive disorder were the most predictive of suicidal ideation in both sexes. The odds ratio for suicidal ideation associated with having 3 comorbid disorders was 2.70 in males and 3.06 in females.	II	B
S. Mazloomzadeh, S. Biglari and F. Eskandari	2021	Mental health, quality of life and their related factors among elderly people in Zanjan health centers, 2017	Cross-sectional study was - standardized questionnaires	To determine the mental health status, QOL, and their related factors in the elderly referred to health centers in Zanjan, Iran in 2017.	There was a significant relationship between mental health and sex, education, occupation, and income. Moreover, a significant association was observed between the QOL and variables such as age, education, occupation, type of residence,	II	B

Authors	Year	Title	Design	Aim	Findings	Evidence Levels	Quality Ratings
					and income. In addition, there was a significant correlation between all domains of QOL and mental health.		
Mokhtari, A. M., Sahraian, S., Hassanipour, S., Baseri, A., Mirahmadizadeh, A.	2019	The epidemiology of suicide in the elderly population in Southern Iran	Cross-sectional study- Mental Health and Suicide Surveillance Systems of Fars province	To investigate the suicide rates among persons 65 years and above in Fars, Iran.	The rate of suicide attempts and deaths were 21.47 and 4.52 per 100,000 population, respectively; and the case fatality rate (CFR) of suicide was 21.07% during the study period. Considering the increasing trend of suicide rates in the elderly in Fars, Iran, measures should be taken to facilitate the access of this group to the mental health care system and counseling.	II	B
S. K. Malakouti, A. K. Akhlaghi, F. Shirzad, V. Rashedi, M. Khlafbeigi, M. M. Lakeh, et al.	2021	Urban mental health initiative: Developing interdisciplinary collaboration, the role of the family physician in management of severe mentally ill patients	Reviewing literature, and using the opinions of national and international experts	To provide a practical model for mental health services in patients with severe psychiatric disorders in urban areas.	To achieve the desired model of urban mental health services so that it can provide comprehensive services to patients at different levels from prevention and care to treatment and rehabilitation, there is a need for collaboration between organizations providing these services.	III	B
Shahmohammadi S., Yaghobi H., Bolhari J., Moshirpour S.	2013	Integration of mental health care in primary health care in Iran: A systematic review	Systematic review	To provide a systematic review of studies conducted between 1998 and 2008 on the primary healthcare program (PHC) in Iran.	The integration of mental healthcare in PHC program has been successful in smaller cities and rural areas, but it has not been responsive to populations of large metropolitan cities in Iran.	III	B
